# Mechanistic insights into the ATP-mediated and species-dependent inhibition of TrpRS by chuangxinmycin†

**DOI:** 10.1039/d5cb00060b

**Published:** 2025-05-09

**Authors:** Yichen Ren, Sili Wang, Wen Liu, Jing Wang, Pengfei Fang

**Affiliations:** a State Key Laboratory of Chemical Biology, Shanghai Institute of Organic Chemistry, University of Chinese Academy of Sciences, Chinese Academy of Sciences 345 Lingling Road Shanghai 200032 China wliu@sioc.ac.cn jwang@sioc.ac.cn fangpengfei@sioc.ac.cn; b School of Chemistry and Materials Science, Hangzhou Institute for Advanced Study, University of Chinese Academy of Sciences, 1 Sub-lane Xiangshan Hangzhou 310024 China

## Abstract

Chuangxinmycin (CXM) is a promising antimicrobial compound targeting bacterial tryptophanyl-tRNA synthetase (TrpRS), an essential enzyme in protein synthesis. The detailed inhibitory mechanism of CXM, particularly in clinically relevant pathogenic bacteria, is poorly understood. In this study, based on the determination of 10 crystal structures, including *Escherichia coli* TrpRS (*Ec*TrpRS) and *Staphylococcus aureus* TrpRS (*Sa*TrpRS) in complex with CXM, ATP, tryptophan, or CXM derivatives, either individually or in combination, as well as the structure of apo-*Sa*TrpRS, we provide key insights into the binding mode of CXM and its species-specific inhibitory mechanisms. Combined with molecular dynamics simulations and binding energy analysis, we demonstrate that CXM binds to *Ec*TrpRS in a manner highly similar to the natural substrate tryptophan. Key residues, including D135 and Y128, play critical roles in CXM recognition and fixation, while conserved hydrophobic residues contribute significantly to binding free energy. This binding pattern is consistent with that observed in *Geobacillus stearothermophilus* TrpRS (*Gs*TrpRS). However, *Sa*TrpRS exhibits distinct behavior due to structural differences, particularly the orientation of Y126 (corresponding to Y128 in *Ec*TrpRS). This difference results in the selectivity of 3-methylchuangxinmycin (mCXM), a CXM derivative, against *Sa*TrpRS. Furthermore, modeling CXM into the tryptophan-binding site of human cytoplasmic TrpRS (*Hs*TrpRS) reveals the lack of key hydrogen bonds and a salt bridge interaction, which likely underlies CXM's significantly weaker inhibition of *Hs*TrpRS. These findings deepen our understanding of the inhibitory mechanism of CXM and its selectivity toward bacterial TrpRSs, and thus can facilitate the design of next-generation antibiotics targeting bacterial TrpRSs.

## Abstract 2

Multidimensional structural and mechanistic analyses elucidate the molecular basis of chuangxinmycin's species-selective inhibition of bacterial tryptophanyl-tRNA synthetases.

## Graphical abstract

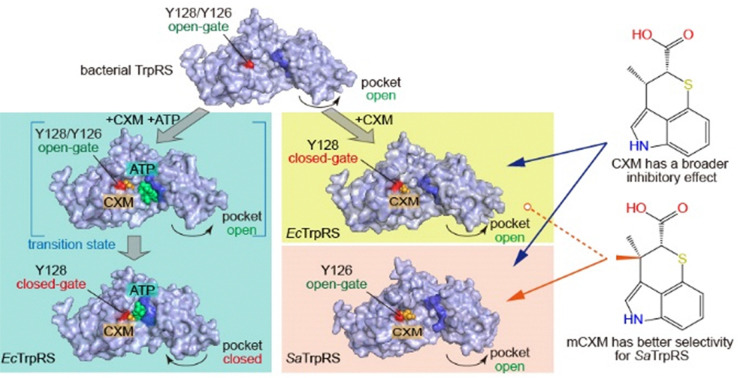

## Introduction

The prolonged and widespread use of antimicrobials has led to the rapid accumulation of drug resistance in pathogenic microorganisms over the past few decades.^1^ This phenomenon poses a significant threat to global public health, as many conventional antibiotics are becoming increasingly ineffective against resistant pathogens. The scarcity of new antimicrobial developments exacerbates the challenge of treating infections caused by rapidly emerging drug-resistant pathogens. Therefore, there is an urgent need to discover and develop innovative antimicrobial agents that can effectively target resistant pathogens and overcome existing resistance mechanisms.^2^

**Fig. 1:**
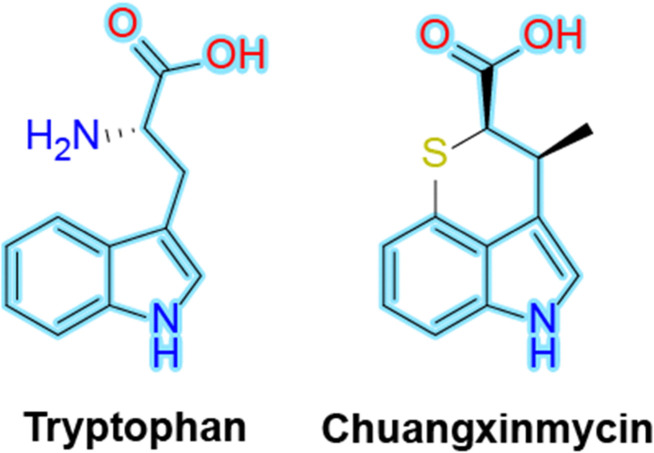
Chemical structures of tryptophan and chuangxinmycin. The shared indole-3-propionic acid core highlighted in light blue.

In the search for new antimicrobials, it is found that natural products have historically been a rich source of bioactive compounds. Chuangxinmycin (CXM), a natural product isolated from *Actinoplanes tsinanensis* CPCC 200056, represents a promising candidate in this regard.^3^ CXM has demonstrated potent *in vitro* antibacterial activity against both Gram-positive and Gram-negative bacteria including clinically relevant pathogens such as *Escherichia coli* and *Staphylococcus aureus*.^4^ Structurally, CXM is a tryptophan (Trp) mimic but is characterized by a unique dihydrothiopyran heterocycle, which distinguishes it from other known antibiotics and contributes to its distinct physicochemical properties (Fig. 1). Its small size and hydrophobic surface further enhance its potential as a drug-like molecule. Additionally, the clarification of the biosynthetic pathway of CXM allows the development of bioretrosynthesis or semisynthesis of CXM or its analogues.^5–7^ These features, combined with its low toxicity and low cross-resistance with common antibiotics, make CXM a compelling subject for further research and development.

The antibacterial activity of CXM is mediated through its inhibition of tryptophanyl-tRNA synthetase (TrpRS), an essential enzyme in the protein translation machinery of bacteria. TrpRS catalyzes the ligation of Trp to its cognate tRNA, forming tryptophanyl-tRNA (Trp-tRNA), which is required for the accurate translation of genetic codes into proteins.^8,9^ As a member of the aminoacyl-tRNA synthetase (aaRS) family, TrpRS plays a critical role in maintaining the fidelity of protein synthesis and is indispensable for bacterial survival and growth. Importantly, bacterial TrpRS exhibits significant structural and functional differences compared to its mammalian counterpart, making it an attractive target for the development of selective antibiotics. CXM has been shown to selectively inhibit bacterial TrpRS with minimal activity against mammalian cytoplasmic TrpRS,^10^ highlighting its potential as a targeted therapeutic agent.

Despite its promising antibacterial properties, the detailed mechanistic understanding of CXM is incomplete. Therefore, the derivatization of CXM is limited by the lack of structural guidance. Most of the synthesized CXM analogues showed poor activity compared with CXM.^4,10,11^ While the crystal structure of CXM in complex with TrpRS from *Geobacillus stearothermophilus* (*Gs*TrpRS) has been determined,^12^ some key aspects of its inhibitory mechanism remain unresolved. For instance, the interactions between CXM and ATP within the active site of TrpRS, as well as the structural basis for its selectivity between bacterial and mammalian TrpRS, have not been fully elucidated. Additionally, the binding behavior of CXM to TrpRS from clinically relevant pathogens, such as *E. coli* and *S. aureus*, has not been thoroughly investigated. These knowledge gaps hinder the rational optimization of CXM and the development of more potent TrpRS-targeting antibiotics.

In this study, we aim to address these limitations by determining high-resolution crystal structures of CXM in complex with TrpRS from *E. coli* (*Ec*TrpRS) and *S. aureus* (*Sa*TrpRS), including complexes with ATP. These structures will provide detailed insights into the binding mode of CXM and its interactions with key residues and cofactors within the active site of TrpRS. Furthermore, we employ molecular dynamics simulations to explore the dynamic behavior of CXM binding and to elucidate the structural determinants of its selectivity for bacterial TrpRS over the mammalian enzyme. By uncovering the mechanistic details of CXM inhibition of TrpRS, this study aims to contribute to the development of next-generation antibiotics targeting this essential bacterial enzyme.

## Results and discussion

### Overall structure of *Ec*TrpRS/CXM and *Sa*TrpRS/CXM complexes

**Fig. 2:**
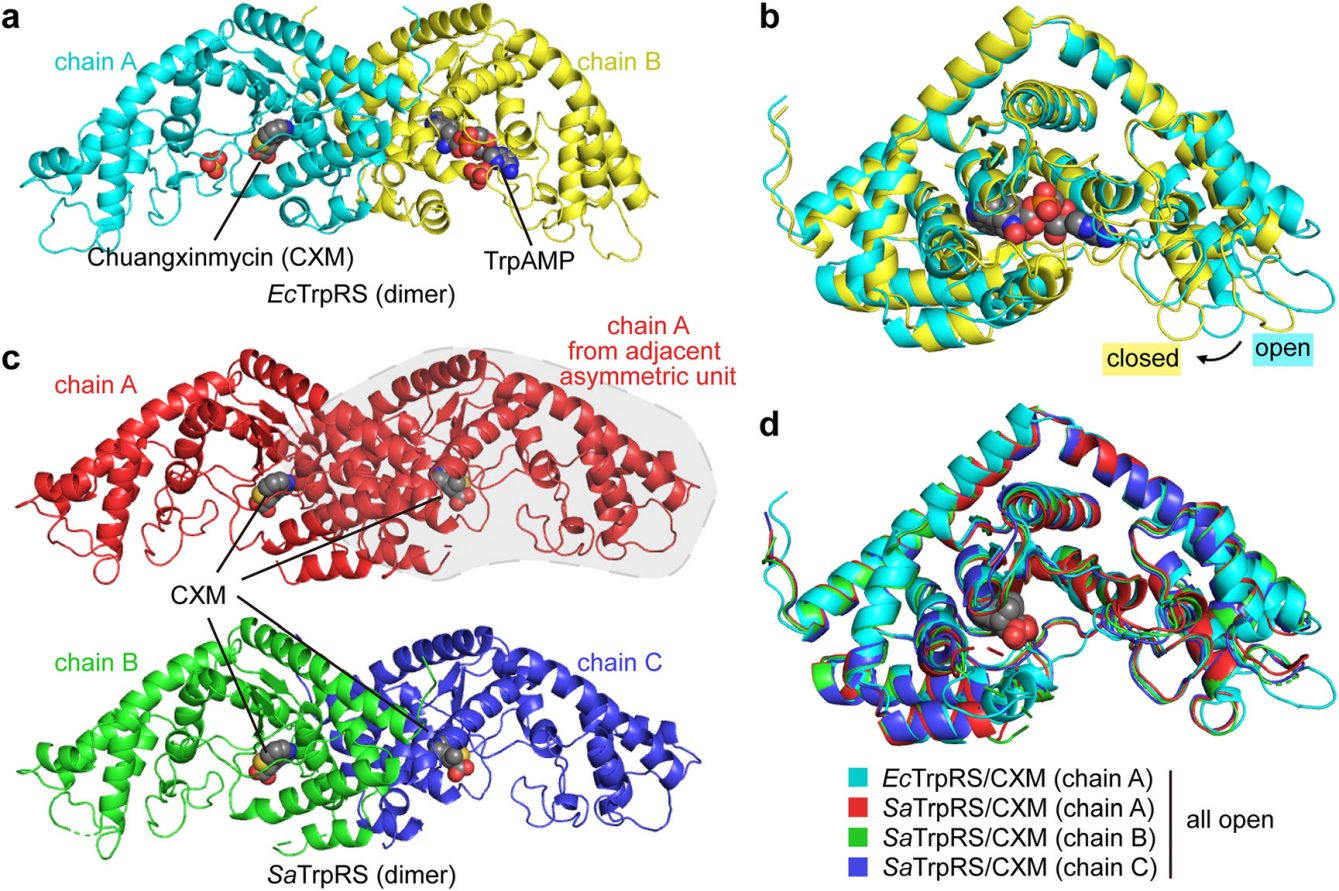
Overall structures of *Ec*TrpRS and *Sa*TrpRS complexes. (a) The asymmetric unit of the *Ec*TrpRS complex structure comprises two chains: chain A bound to a CXM molecule and chain B bound to an endogenous tryptophanyl-5′-AMP (TrpAMP) molecule. (b) The *Ec*TrpRS/CXM complex (chain A) adopts an open conformation, while the *Ec*TrpRS/TrpAMP complex (chain B) adopts a closed conformation. (c) The asymmetric unit of the *Sa*TrpRS/CXM crystal structure contains three chains, each bound to a CXM molecule. (d) Conformational comparison of the *Ec*TrpRS/CXM and *Sa*TrpRS/CXM complexes reveals that all chains adopt an open conformation.

To elucidate the structural details of CXM binding to TrpRS, we determined the crystal structures of *Ec*TrpRS and *Sa*TrpRS in complex with CXM. The structure of the *Ec*TrpRS complex was solved at a resolution of 2.24 Å (Table S1, ESI†). Its asymmetric unit contains two chains in the open and closed states respectively (Fig. 2a and b). Chain A exists in an open state and binds to a CXM molecule (denoted as the *Ec*TrpRS/CXM structure, Fig. S1a, ESI†), whereas chain B is in a closed state and binds to an endogenous tryptophanyl-5′-AMP (TrpAMP) molecule (denoted as *Ec*TrpRS/TrpAMP structure, Fig. S1b, ESI†), which is similar to the previously reported structures of *Ec*TrpRS complexes (PDB: 8I1W and 8I4I).^13^ The structure of the *Sa*TrpRS complex was solved at resolutions of 2.38 Å (Table S1, ESI†). Its asymmetric unit contains three chains (A, B and C), each binding a CXM molecule (Fig. S1c, ESI†). The chains B and C form a dimer and chain A forms a dimer with another chain A of an adjacent asymmetric unit (Fig. 2c and Fig. S2, ESI†). To ensure rigorous structural comparative analysis, we also determined the structures of *Sa*TrpRS in the absence of substrates or inhibitors (apo-*Sa*TrpRS) and *Sa*TrpRS in complex with Trp (*Sa*TrpRS/Trp complex) at resolutions of 3.04 Å and 2.32 Å, respectively (Table S2 and Fig. S3, ESI†). These structures share a similar three-chain asymmetric unit with the *Sa*TrpRS/CXM complex.

The CXM complexes show high conformational similarity to Trp complexes and apo proteins (Fig. S4a, ESI†). The superimposition of the *Ec*TrpRS/CXM structure with the *Ec*TrpRS-apo structure (PDB: 8I1W chain A) and the *Ec*TrpRS/Trp structure (PDB: 8I4I chain A) yielded root mean square deviation (RMSD) values of 0.4421 Å and 0.4172 Å, respectively. Similar superimposition of *Sa*TrpRS structures (chain A) gave RMSD values of 0.3660 Å and 0.2868 Å (Fig. S4b, ESI†).

Therefore, the CXM complexes of both *Ec*TrpRS and *Sa*TrpRS are in the open state (Fig. 2d), similar to the Trp complexes. Notably, a previous study reported a closed pre-transition state (PreTS) *Gs*TrpRS/CXM complex structure (PDB: 7CMS, chain B), revealing an inward movement of the KMSKS motif-containing loop, which suggests a conformational transition induced by CXM binding.^12^ However, no similar movement was observed in the *EcT*rpRS/CXM or *Sa*TrpRS/CXM complexes (Fig. S5, ESI†). These findings suggest that the PreTS conformational transition triggered by CXM in *Gs*TrpRS may not be conserved across TrpRS from different species, implying the existence of species-specific conformational transitions during CXM binding. This phenomenon may be linked to the species-selective behavior of CXM.

### Binding mode of CXM and *Ec*TrpRS or *Sa*TrpRS

**Fig. 3:**
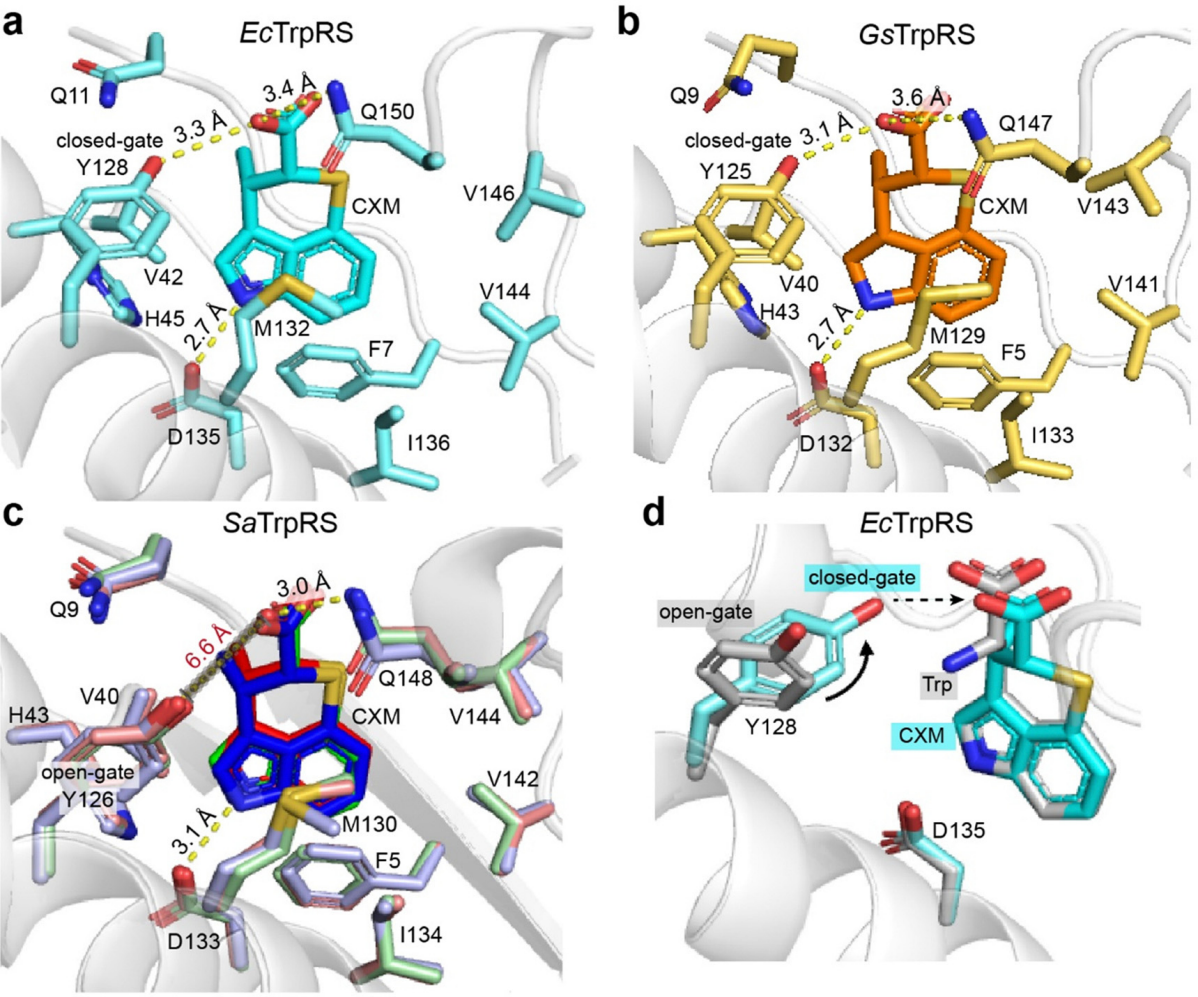
Interactions between CXM and TrpRS across species. (a) *Ec*TrpRS/CXM complex. (b) *Gs*TrpRS/CXM (PDB: 7CMS, chain A). (c) *Sa*TrpRS/CXM, with chains A, B, and C colored red, green, and blue, respectively. The distance between Y126 and CXM carboxylate (6.6 Å) indicates the lack of hydrogen bond with Y126. (d) Structural comparison of the *Ec*TrpRS/CXM complex (cyan) and the *Ec*TrpRS/Trp complex (gray; PDB: 8I4I, chain A). In the CXM complex, Y128 adopts a “closed-gate” conformation, with its phenol group oriented toward the ligand in the Trp-binding pocket. In contrast, Y128 in the Trp complex adopts an “open-gate” conformation, with its phenol group oriented away from the Trp-binding pocket.

The interaction between CXM and *Ec*TrpRS is similar to that in the open state of the *Gs*TrpRS/CXM complex (PDB: 7CMS chain A). Specifically, the nitrogen atom of the indolyl group of CXM forms a hydrogen bond with the carboxyl group of D135 (corresponding to D132 in *Gs*TrpRS, Fig. S6, ESI†), and the carboxyl group of CXM forms a hydrogen bond with the phenol group of Y128 (corresponding to Y125 in *Gs*TrpRS, Fig. S6, ESI†) in both structures (Fig. 3a and b). In addition, the carboxyl group of CXM also forms a hydrogen bond with the residue Q150 of *Ec*TrpRS (3.4 Å) and has a polar interaction with the corresponding Q147 in *Gs*TrpRS (3.6 Å) (Fig. 3a and b).

As for the *Sa*TrpRS/CXM complex, the binding sites and orientations of CXM molecules in all the three chains are highly similar to each other, and are also similar to those in *Ec*TrpRS and *Gs*TrpRS complexes (Fig. 3c). However, the detailed interactions between CXM and *Sa*TrpRS differ slightly. For example, the residue Q148 (corresponding to Q150 in *Ec*TrpRS, Fig. S6, ESI†) is closer to the CXM carboxyl group (3.0 Å in chain A), resulting in a stronger interaction (Fig. 3c). The phenol group of Y126 in *Sa*TrpRS (corresponding to Y128 in *Ec*TrpRS, Fig. S6, ESI†) adopts a distinct sidechain orientation, positioning it farther from the CXM carboxyl group. Notably, this orientation aligns with that of Y128 in the Trp-bound state of both *Sa*TrpRS and *Ec*TrpRS (Fig. 3d and Fig. S4c, ESI†).

Significant hydrophobic interactions are observed in all three bacterial TrpRSs (*Ec*TrpRS, *Sa*TrpRS, and *Gs*TrpRS) during CXM binding. Conserved residues F7, V42, H45, M132, I136, V144, and V146, located within 4 Å of the CXM carbon skeleton, engage in hydrophobic or π-stacking interactions with CXM (Fig. 3 and Fig. S6, ESI†). CXM not only mimics the natural substrate Trp but also adopts a closed-ring structure that fixes the carboxyl group in a similar orientation to Trp (Fig. 3d). This conformational locking likely stabilizes the binding of CXM to TrpRS and may enhance its binding affinity compared to the more flexible natural substrate.

In summary, CXM binds to *Ec*TrpRS and *Sa*TrpRS in a manner highly similar to its interaction with *Gs*TrpRS, with key hydrogen bonds and hydrophobic interactions stabilizing the complex. While the overall binding mode is conserved, subtle differences in residue positioning and interaction strength highlight the nuanced adaptability of CXM to different TrpRS variants. Importantly, CXM not only mimics Trp but also locks its binding conformation, potentially enhancing its binding affinity and making it a potent competitive inhibitor in bacterial protein synthesis.

### MD simulations reveal different binding behaviors of CXM in the *Ec*TrpRS and *Sa*TrpRS systems

To further study the dynamic properties of CXM binding, molecular dynamics (MD) simulations were performed, and the MM/GBSA method was used to calculate the binding free energy.^14^ The RMSD of the *Ec*TrpRS system stabilized after 15 ns of simulation (Fig. S7a, ESI†), whereas the *Sa*TrpRS system required an additional 20 ns to converge, with the last 5 ns of the trajectory used for detailed analysis (Fig. S7b and c, ESI†).

**Fig. 4:**
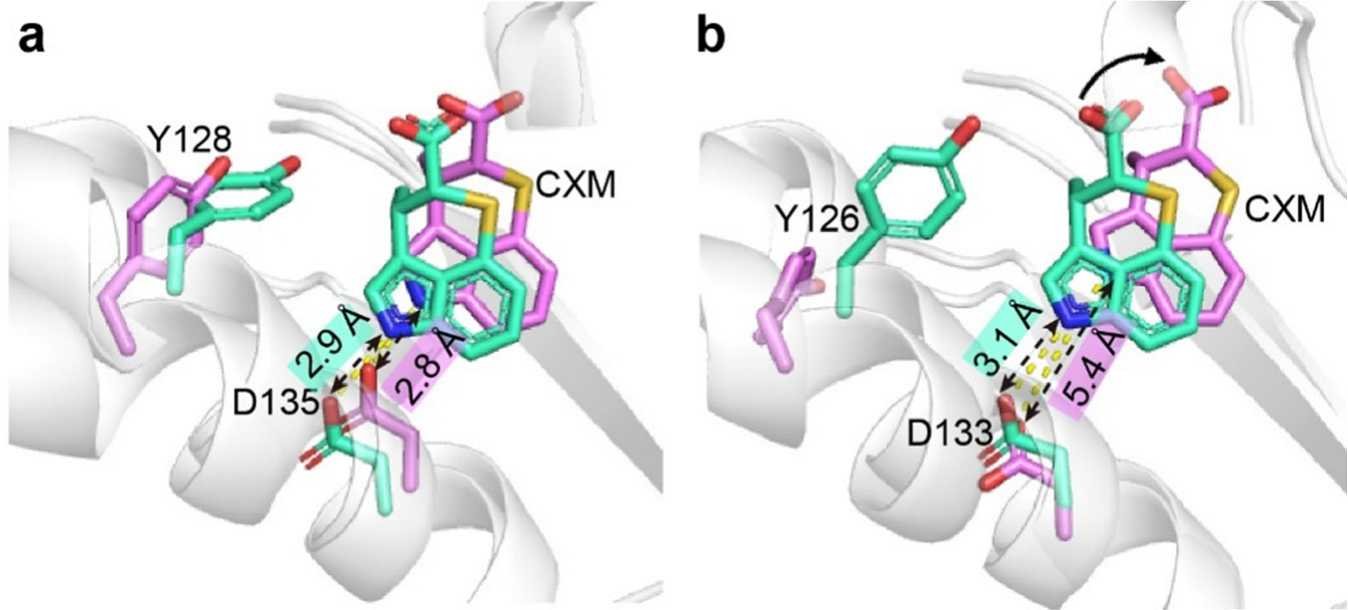
Superimposition of the pocket and ligand structures before (green-cyan) and after (pink-purple) MD simulations. (a) *Ec*TrpRS/CXM system. (b) *Sa*TrpRS/CXM system.

After simulation, the ligand-binding pose in *Ec*TrpRS/CXM system remained similar to the initial structure (Fig. 4a), with the hydrogen bond between the indolyl nitrogen atom and D135 carboxylate preserved. Hydrophobic interactions of CXM with F7, V42, M132, I136, V144, and V146, as well as the polar interaction with Q150, were confirmed by binding free energy decomposition and virtual alanine scanning (Table 1). These results align with those observed in the *Gs*TrpRS/CXM system.^12^

**Table 1:** Contribution of key residues in the pocket to the binding free energy of CXM (kcal mol^−1^), evaluated by free energy decomposition (FED) and virtual alanine scanning (AS)

*Ec*TrpRS			*Sa*TrpRS		
Residue	FED	AS	Residue	FED	AS
F7	−0.78	−1.02	F5	−0.72	−1.52
Q11	−0.34	−0.40	Q9	−1.52	−1.22
V42	−1.04	−1.62	V40	−1.00	−1.62
H45	−0.50	−2.03	H43	−0.01	−0.49
Y128	0.01	−0.06	Y126	0.01	−0.05
M132	−0.34	−0.85	M130	−0.23	−0.61
D135	0.63	−2.13	D133	0.20	0.20
I136	−0.32	−0.67	I134	−0.52	−1.11
V144	−0.87	−0.96	V142	−0.62	−0.95
V146	−0.64	−0.87	V144	−1.57	−1.65
Q150	−1.80	−3.21	Q148	−2.78	−5.10

In contrast, the *Sa*TrpRS/CXM system exhibited different behavior during simulations. The orientation of CXM changed, and the indolyl nitrogen atom of CXM moved away from the hydrogen bonding location of D133 (Fig. 4b). The binding free energy analysis confirmed the loss of interaction with D133, while the hydrophobic interactions and remained unchanged. The “open-gate” orientation of Y126 in the *Sa*TrpRS/CXM system likely allows greater mobility of the CXM molecule (Fig. 3c), as suggested by previous research on the role of tyrosine residues in limiting ligand movement.^15–17^

Therefore, the molecular dynamics simulations and binding free energy analysis revealed distinct binding behaviors of CXM in the *Ec*TrpRS and *Sa*TrpRS systems. While CXM maintained its initial binding pose and key interactions in *Ec*TrpRS, its orientation shifted in *Sa*TrpRS, leading to the loss of hydrogen bonding with D133 while retaining hydrophobic interactions. These findings provide key insights into the binding mechanisms of CXM with different TrpRS systems.

### Structure of *Ec*TrpRS/CXM/ATP complexes

**Fig. 5:**
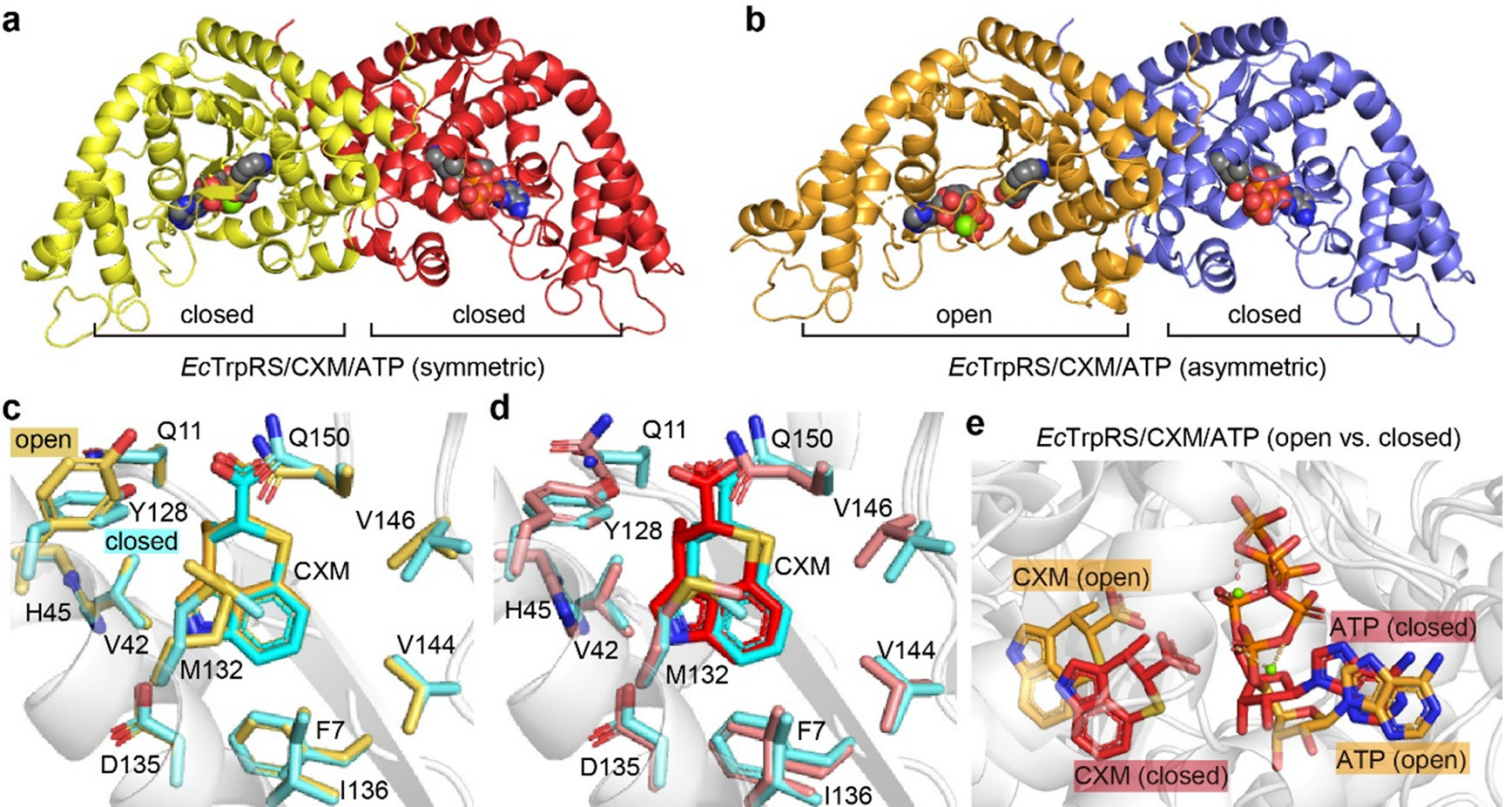
Structural features of two forms of *Ec*TrpRS/CXM/ATP complexes. (a) Symmetric closed–closed *Ec*TrpRS/CXM/ATP complex structure. (b) Asymmetric open–closed *Ec*TrpRS/CXM/ATP complex structure. (c) CXM binding comparison between the open-state *Ec*TrpRS/CXM/ATP subunit (orange) and the *Ec*TrpRS/CXM subunit (cyan). (d) CXM binding comparison between the closed-state *Ec*TrpRS/CXM/ATP structure (red, subunit B of the symmetric complex crystallized with ATP) and the *Ec*TrpRS/CXM structure (cyan, crystallized without ATP). (e) Superimposition of the open-state (orange) and closed-state (red) *Ec*TrpRS/CXM/ATP.

To explore the effect of ATP on CXM binding, we further conducted crystallographic studies of the structure of the *Ec*TrpRS/CXM/ATP complex. Two different structures were determined at resolutions of 1.92 Å and 2.15 Å (Table S3, ESI†). Both structures contain two chains in the asymmetric unit, each binding a CXM molecule, an ATP molecule and a Mg^2+^ ion (Fig. 5a, b and Fig. S8, ESI†). However, one structure, which is symmetric, comprises two chains in a similarly closed conformation with an RMSD value of 0.7758 Å (Fig. S9a, ESI†). In contrast, the other structure, which is asymmetric, features two chains in markedly different conformations (with an RMSD value of 2.6220 Å), with one chain in an open state and the other in a closed state (Fig. S9b, ESI†).

In the asymmetric structure, the open-state chain A closely resembles the conformation of the *Ec*TrpRS/CXM complex without ATP (RMSD value of 0.6189 Å), except for a distinct shape in the region 107–120 (Fig. S10a, ESI†). The binding pose and interactions of CXM are similar to those in the *Ec*TrpRS/CXM complex (Fig. 5c). Interestingly, the orientation of Y128 adopts an “open-gate” state (Fig. 5c), resembling the apo or Trp-bound TrpRS structures, rather than the “closed-gate” state observed in the *Ec*TrpRS/CXM complex or the closed-state *Ec*TrpRS/CXM/ATP structures (Fig. 5d). In this structure, ATP binding resembles that in the open-state *Gs*TrpRS/ATP complex (PDB: 1MAW) (Fig. S10b, ESI†).

The other three closed-state structures exhibit conformations similar to the *Ec*TrpRS/TrpAMP complex (RMSD values of 0.96–1.00 Å). Compared to the *Ec*TrpRS/CXM complex, the CXM molecules in these subunits show slight rotational differences (Fig. 5d). The ATP binding mode in the closed state of the *Ec*TrpRS/CXM/ATP structure is also different from that in the open state structure (Fig. 5e). Instead, in these closed state structures, ATP binding is similar to that in the PreTS *Gs*TrpRS/tryptophanamide/ATP complex (PDB: 1MAU) or TrpRS/TrpAMP complexes (Fig. S10c, ESI†).

Together, this analysis highlights the conformational flexibility of *Ec*TrpRS upon ATP binding. The presence of ATP does not preclude the binding of CXM, and it has a high likelihood of cooperating with CXM to induce TrpRS to adopt a closed conformation, potentially synergistically enhancing the inhibitory effect of CXM on TrpRS.

### Tolerance of the TrpRS pocket to CXM analogues

**Fig. 6:**
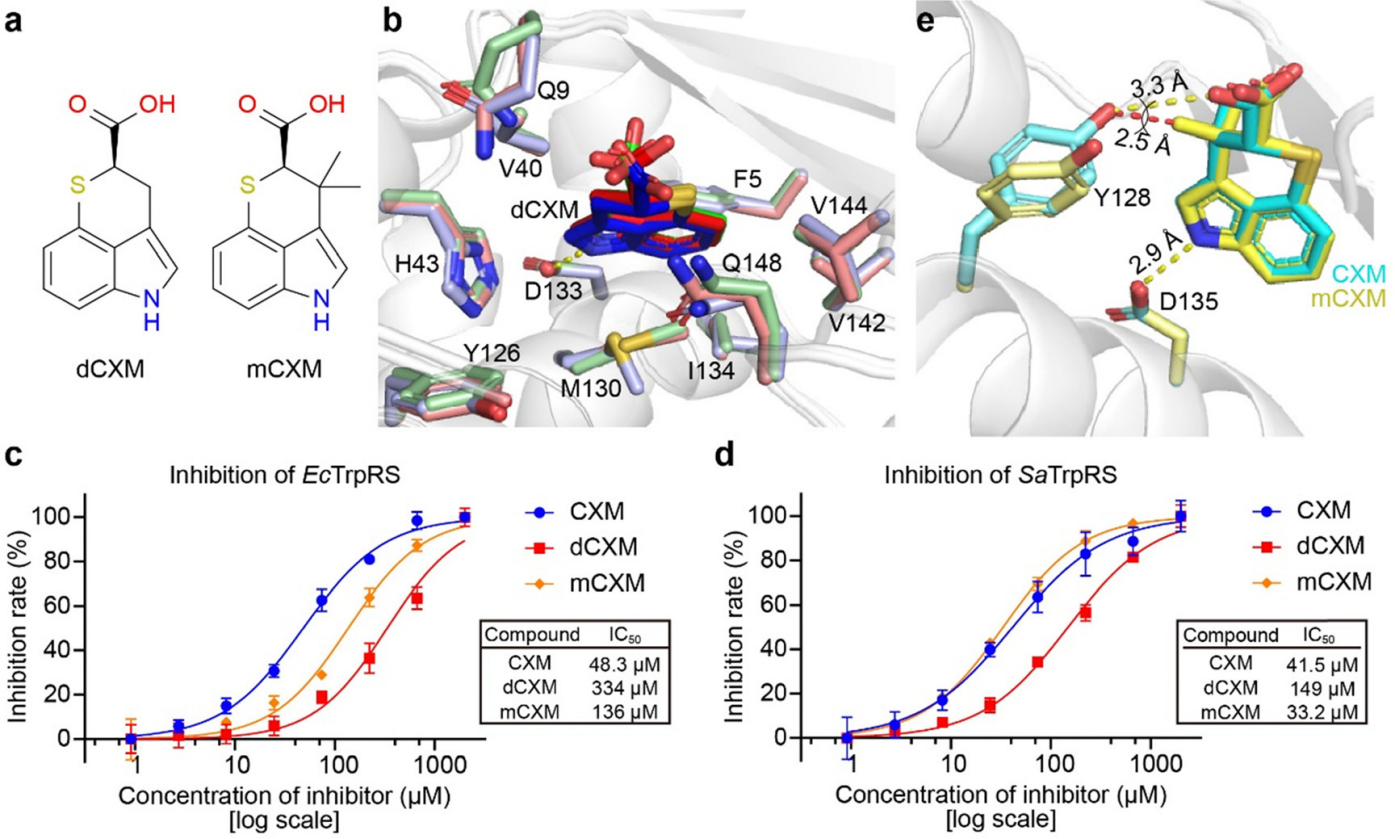
Binding and inhibitory activity of dCXM and mCXM. (a) Chemical structures of dCXM and mCXM. (b) Binding of dCXM in three chains within the asymmetric unit of the *Sa*TrpRS/dCXM complex. The protein main chain is shown as gray cartoons, with dCXM and key interacting residues displayed as sticks. (c) Inhibition of *Ec*TrpRS by CXM, dCXM, and mCXM, as measured by ATP hydrolysis assays. (d) Inhibition of *Sa*TrpRS by CXM, dCXM, and mCXM, as measured by ATP hydrolysis assays. (e) Structural comparison of *Ec*TrpRS complexed with mCXM and CXM. Y128, D135, and ligands (mCXM or CXM) are shown as sticks (yellow for mCXM complex, cyan for CXM complex). The protein main chain is displayed as gray cartoons. The additional methyl group in mCXM induces repulsion, forcing Y128 into an “open-gate” conformation in the mCXM-bound structure.

To investigate the tolerance of the TrpRS pocket, two analogues of CXM (Fig. 6a), 3-demethylchuangxinmycin (dCXM) and 3-methylchuangxinmycin (mCXM), were tested. The complex structures of these analogues with *Ec*TrpRS and *Sa*TrpRS were solved. All analogue complexes are isomorphous with the corresponding CXM complexes (Tables S4 and S5, ESI†), and the binding modes are nearly identical (Fig. 6 and Fig. S11, S12, ESI†).

The flexibility of dCXM leads to diverse conformations across the three chains of the *Sa*TrpRS/dCXM complex (Fig. 6b). Consistent with its more flexible conformation and reduced hydrophobic surface area, dCXM demonstrates significantly lower inhibitory activity compared to CXM (Fig. 6c and d).

The inhibitory activity of mCXM against *Ec*TrpRS is lower than that of CXM, likely due to steric hindrance from the additional methyl group, which prevents the “closed-gate” conversion of Y128 (Fig. 6c and e). Interestingly, mCXM exhibits stronger inhibition of *Sa*TrpRS compared to *Ec*TrpRS (Fig. 6c and d), and its activity against *Sa*TrpRS is not weaker than that of CXM (Fig. 6d), suggesting that its binding to SaTrpRS is independent of the stabilization of the closed Y126 “gate”.

These findings highlight the importance of structural flexibility and steric effects in ligand binding to TrpRS. The differential inhibitory activities of dCXM and mCXM against *Ec*TrpRS and *Sa*TrpRS suggest species-specific adaptations in the TrpRS binding pocket, which could provide information in the design of more selective inhibitors, which is particularly important in the context of antibiotic development to minimize off-target effects on host cells and non-target microbial communities, thereby reducing the risk of resistance.

### Mechanistic insights into the ATP-mediated and species-dependent inhibition of TrpRS by CXM

In this work, two distinct states of the *Ec*TrpRS/CXM/ATP ternary complex were observed (Fig. 5). These states may reflect two stages of CXM binding to *Ec*TrpRS/ATP, analogous to the structural transformations of *Gs*TrpRS during Trp and ATP binding.^15–17^ Based on these observations, we propose that the inhibitory mechanism of CXM against TrpRS is both ATP-mediated and species-dependent.

Under high ATP concentrations, CXM, a conformational mimic of Trp, initially binds to the open-state *Ec*TrpRS/ATP complex, forming an open-state *Ec*TrpRS/CXM/ATP intermediate. Subsequently, the Y128 “gate” closes, transitioning the complex to a PreTS state. However, due to the rigidity of CXM and the stabilization of its carboxylate anion by a hydrogen bond with Y128, the nucleophilic attack on ATP is blocked, trapping the complex in a transition state-like conformation.

In contrast, under lower ATP concentrations, CXM alone can induce the “closed-gate” state of Y128 in the *Ec*TrpRS/CXM complex (Fig. 3a), suggesting an alternative inhibitory phase. However, this mechanism is not conserved across bacterial species. For instance, in the *Sa*TrpRS/CXM complex, Y126 remains in an “open-gate” state, and CXM is not stably fixed during MD simulations. Additionally, in the *Gs*TrpRS/CXM complex (PDB: 7CMS), there is another alternative inhibitory phase, where CXM binding induces a PreTS conformational transition.^12^

These differences highlight the complexity of CXM's inhibitory mechanism and suggest that the response of TrpRS/CXM systems to ATP concentration may vary among bacterial species, potentially influencing their *in vivo* sensitivity to CXM. Further biochemical and antibacterial studies are needed to elucidate these species-specific differences in detail.

### Selectivity of CXM for bacterial TrpRS over human TrpRS

**Fig. 7:**
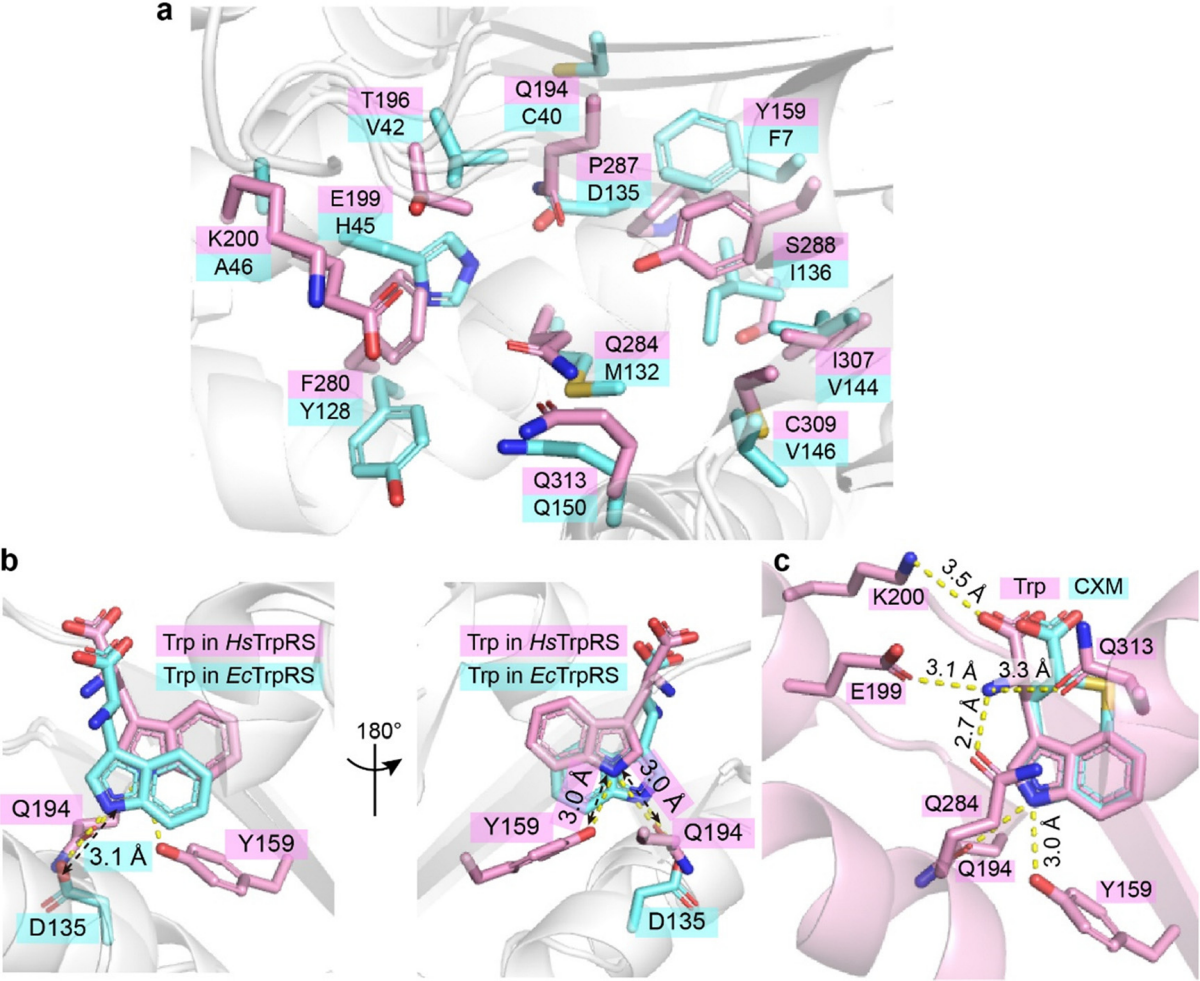
Differences in substrate binding mechanisms between *Hs*TrpRS and *Ec*TrpRS. (a) A detailed comparison of the key residues within the Trp-binding pocket of *Hs*TrpRS (pink) and *Ec*TrpRS (cyan) reveals significant differences. The conformationally equivalent residues in both enzymes, which play crucial roles in substrate binding, are labeled for clarity. (b) The Trp binding sites in *Hs*TrpRS and *Ec*TrpRS exhibit notable variations in structure and composition. (c) When the CXM molecule (depicted in cyan sticks) from the *Ec*TrpRS complex is modeled into the Trp binding site of *Hs*TrpRS (pink), there is a notable absence of effective hydrogen bonds, with the exception of one formed by the indolyl nitrogen of CXM.

CXM is a selective inhibitor of bacterial TrpRSs, exhibiting significantly weaker inhibition of human cytoplasmic TrpRS (*Hs*TrpRS). To explore the molecular basis of this selectivity, CXM was modeled into the Trp-binding pocket of *Hs*TrpRS by superimposing its indolyl group with that of Trp (PDB: 2QUH). Despite the absence of notable steric hindrance, the interaction patterns between CXM and HsTrpRS differed substantially from those observed with bacterial TrpRSs, primarily due to the nearly complete divergence of key residues within the Trp-binding pocket (Fig. 7).

The Trp-binding pocket of *Hs*TrpRS is more hydrophilic compared to that of bacterial TrpRSs (Fig. 7a). In *Hs*TrpRS, key hydrogen bonds formed by residues E199, Q284, and Q313 with the amino group of Trp play a crucial role in ligand recognition. In contrast, although the carboxyl group of CXM can form a hydrogen bond with Y128 in *Ec*TrpRS, its orientation and charge are incompatible with similar interactions in *Hs*TrpRS (Fig. 7c). Additionally, while the carboxyl group of Trp forms a salt bridge with K200 in *Hs*TrpRS, the carboxyl group of CXM is positioned too far from K200 to establish any interaction (Fig. 7c). These observations suggest that the lack of key polar interactions—such as hydrogen bonds and salt bridges—is a major factor contributing to the rejection of CXM by *Hs*TrpRS.

This selectivity of CXM for bacterial TrpRSs over *Hs*TrpRS aligns with its proposed inhibitory mechanism, which relies on specific interactions with bacterial TrpRS residues (*e.g.*, Y128 in *Ec*TrpRS and some hydrophobic residues) and the stabilization of a transition state-like conformation. The inability of CXM to form analogous interactions in *Hs*TrpRS further underscores its potential as a species-selective antibacterial agent.

## Conclusions

This study provides a comprehensive structural and mechanistic understanding of how chuangxinmycin (CXM) inhibits bacterial tryptophanyl-tRNA synthetases (TrpRSs), offering valuable insights into its binding mode, conformational dynamics, and species-specific selectivity.

CXM represents a distinctive single-site aaRS inhibitor that selectively targets bacterial TrpRS by competitively occupying the tryptophan-binding pocket without excluding other substrates from binding (Fig. S13, ESI†).^18^ This mechanism is analogous to resveratrol inhibition of tyrosyl-tRNA synthetase (TyrRS) by blocking the tyrosine-binding site.^19^ Unlike cladosporin or AN2690, which target different sites (Fig. S13, ESI†),^20–22^ CXM achieves potent and selective inhibition solely through high-affinity engagement of the relatively conserved amino acid-binding pocket, highlighting its unique mechanism among aaRS inhibitors.

Intriguingly, our analyses suggest that CXM's inhibitory efficacy may be enhanced by ATP-induced conformational changes in TrpRS, similar to the ATP-dependent inhibition seen with halofuginone in ProRS.^23^ However, while halofuginone functions as a dual-site inhibitor by simultaneously occupying both the amino acid and tRNA-binding pockets (Fig. S13, ESI†),^24^ CXM currently operates through a single-site mechanism. This distinction presents two promising directions for future optimization: first, by extending CXM's chemical scaffold toward the ATP-binding pocket to create a dual-site inhibitor akin to mupirocin (which mimics Ile-AMP in IleRS),^25^ or second, by engineering interactions with the tRNA-binding pocket while preserving ATP-mediated synergism, thereby emulating halofuginone's strategy but tailored to TrpRS (Fig. S13, ESI†). These approaches could further enhance CXM's inhibitory potency, offering a strategy for developing next-generation antibiotics targeting bacterial TrpRS.

The structural insights gained from this study provide a solid foundation for the rational design of improved TrpRS inhibitors to address the growing challenge of antibiotic resistance.

## Materials and methods

### Cloning of tryptophanyl-tRNA synthetases

The genes encoding the *Sa*TrpRS was codon-optimized, synthesized and cloned into the expression vector pET-28a (+) by TsingKe Biotech. The gene encoding the *Ec*TrpRS was PCR-amplified with Phanta Max Super-Fidelity DNA Polymerase (Vazyme) from the genome of *Escherichia coli* DH-5α strain, cloned into pET-28a (+) by homologous recombination in *E. coli* DH-5α and then checked by sequencing (TsingKe Biotech). The 6× His-tag was added to the C-terminus of both recombinant enzymes. The sequence of genes and primers used in this work is listed in Tables S6 and S7, ESI.†

### Enzyme expression and purification

The recombinant plasmids were transformed into *E. coli* BL21(DE3) strain for protein expression. The transformed cells were cultured in LB medium containing 50 μg mL^−1^ kanamycin at 37 °C and 200 rpm until the optical density at 600 nm (OD600) reached 0.8. Then the overexpression was induced by the addition of 0.3 mM isopropyl β-d-thiogalactoside (IPTG), and the cells were incubated for another 20 h at 16 °C and 200 rpm. Cells were harvested by centrifugation at 5000 rpm for 10 min at 4 °C, resuspended in pre-cooled buffer A (25 mM Tris–HCl, 500 mM NaCl, and 25 mM imidazole, pH 8.0), and disrupted using a high pressure homogenizer (600 bar) in an ice bath. The lysate was centrifuged at 12 000 rpm for 30 min at 4 °C, and the supernatant was loaded on a pre-equilibrated HisTrap HP column (Cytiva) and washed with 9 column volumes of lysis buffer. The protein was eluted by a linear gradient of buffer B (25 mM Tris–HCl, 500 mM NaCl, and 350 mM imidazole, pH 8.0). Fractions containing target protein were collected and diluted to a NaCl concentration lower than 100 mM, loaded on a pre-equilibrated HiTrap Q HP (for *Ec*TrpRS) or HiTrap Heparin HP (for *Sa*TrpRS) column (Cytiva) and washed using 5 column volumes of buffer C (25 mM Tris–HCl, 50 mM NaCl, pH 8.0). The protein was eluted using buffer D (25 mM Tris–HCl, 1 M NaCl, pH 8.0) and further purified using a HiLoad Superdex 200 column (Cytiva) in 25 mM Tris pH 8.0, 200 mM NaCl. The fractions were then collected and concentrated for subsequent experiments.

### Preparation of CXM and its analogues

CXM, dCXM and mCXM used in this work are from our previous research. dCXM is chemically synthesized. CXM and mCXM are separated from a biosynthetic system. All compounds are purified *via* high-performance liquid chromatography (HPLC) and examined using high-resolution mass spectrometry (HR-MS), ^1^H and ^13^C NMR.^7^

### Protein crystallization

The crystallization screening was performed using commercial crystal screen kits (molecular dimensions). Crystallization experiments of *Sa*TrpRS were performed at 18 °C based on the sitting-drop method. The protein was concentrated to 11–16 mg mL^−1^ and the inhibitors were added at a molar ratio of 1 : 4. The crystals of *Sa*TrpRS (apo or complexed with CXM, dCXM or mCXM) were obtained under the conditions of 0.1 M Tris pH 7.8, 5% w/v γ-PGA (Na^+^ form, low molecular) and 20% w/v PEG 2000 MME. Crystallization experiments of *Ec*TrpRS were performed at 4 °C based on the sitting-drop method. The protein was concentrated to 8–11 mg mL^−1^. The inhibitors were added at a molar ratio of 1 : 4 and ATP was added at a molar ratio of 1 : 30 with 2 mM final concentration of MgCl_2_. The crystals of *Ec*TrpRS complexed with TrpAMP and CXM, dCXM or mCXM were obtained from the condition of 0.2 M (NH_4_)_2_SO_4_, 0.1 M MES pH 6.5 and 20% w/v PEG 8000. The crystal of symmetric *Ec*TrpRS/CXM/ATP complex was obtained from the condition of 0.2 M (NH_4_)_2_SO_4_, 0.1 M Bis–Tris pH 6.5 and 25% w/v PEG 3350. The crystal of the asymmetric *Ec*TrpRS/CXM/ATP complex was obtained under the conditions of 0.15 M (NH_4_)_2_SO_4_, 0.1 M sodium citrate pH 5.0, 15% w/v PEG Smear High (containing equal mass of PEG 6000, 8000 and 10 000). All crystals were flash-frozen in liquid nitrogen using the reservoir solution containing 20% v/v glycerol as cryo-protectant before data collection.

### Data collection and structure determination

The X-ray diffraction data were collected at the beamline BL10U2 or BL19U1 of the Shanghai Synchrotron Radiation Facility (SSRF) with a wavelength of 0.979 Å. Data reduction and integration were achieved with XDS^26^ or autoPROC^27^ software suites. The phase problem was solved by molecular replacement method. The starting model of *Sa*TrpRS is predicted by AlphaFold (AF-P67594-F1) and that of *Ec*TrpRS is previously reported (PDB: 8I1W). Iterative cycles of model building and refinement were performed using CCP4,^28^ Coot,^29^ and Phenix.^30^ The structures were analyzed using PyMol (https://www.pymol.org/), and the RMSD values between structures were calculated using secondary structure matches (SSM) in Coot.^31^

### *In vitro* activity assay

The activity of TrpRS was measured *vis* ATP hydrolysis using Kinase-Glo® kit (Promega). The reaction system contains 50 mM KCl, 40 mM MgCl_2_, 100 mM Tris pH 7.5, 0.5 mM dithiothreitol (DTT), 0.004% Tween 20, 0.1 mg mL^−1^ bovine serum albumin (BSA), 2 μM ATP, 10 μM tryptophan, 50 nM TrpRS protein, and 25 nM yeast inorganic pyrophosphatase (recombinant). 50 mM of NH_2_OH was added as a receptor instead of tRNA to release TrpAMP from TrpRS.^32^ The reaction mixture was incubated at 37 °C for 1.5 h (for *Sa*TrpRS) or 3 h (for *Ec*TrpRS), and then mixed with equal volume of detection solution for luminescence measurements. All experiments were performed in three replicates. Data were processed using GraphPad Prism 8.

### Molecular dynamics simulations

The protocol of MD simulations is mainly according to the previous publication.^12^ The cocrystal structures of *Ec*TrpRS and *Sa*TrpRS complexed with CXM got in this research were used as the initial structure of MD simulations. All MD simulations were performed with Amber 20. The net charge of CXM was set to −1 and the partial charges were calculated with Antechamber module in AmberTools 20. The GAFF force field was applied to CXM and the Amber ff14SB force field was applied to the protein. A truncated octahedron solvent box of TIP3P model was added to dissolve the protein, and sodium cations were added to neutralize the system. In energy minimization, all atoms in the protein and CXM were restrained, and the system was minimized for 2000 steps; then restraints on CXM were removed and the system was minimized for another 5000 steps; finally, all the restraints were removed and the system was minimized for 10 000 steps. Then the protein was restrained and the system was heated to 300 K in 50 ps using the NVT ensemble, and all the restraints were removed and the system was equilibrated using the NPT ensemble for 1 ns. In the production phase, the system was simulated for 20 ns using the NPT ensemble and the *Sa*TrpRS system was simulated for another 20 ns to get the RMSD convergence. The trajectory of the last 5 ns (after RMSD convergence) was analyzed using the MM/GBSA method for the binding free energy decomposition and alanine scanning (using the MM/PBSA module in AmberTools 20).

## Author contributions

Investigation and methodology, Y. R., and S. W.; review, editing, and writing, Y. R., S. W., W. L., J. W., and P. F.; conceptualization and supervision, W. L., J. W., and P. F. All authors read and approved the final manuscript.

## Conflicts of interest

The authors declare that they have no competing interests.

## Supplementary Material

CB-006-D5CB00060B-s001

## Data Availability

All crystal structures have been deposited at PDB (https://www.rcsb.org/). The data supporting the conclusion are within the article and its ESI.†
