# Supplementary material for: Mechanistic insights into the ATP-mediated and species-dependent inhibition of TrpRS by chuangxinmycin
Source: RSC Chem Biol. 2025 May 9;6(7):1079–88. doi: 10.1039/d5cb00060b (PMC12093056; doi:10.1039/d5cb00060b)
Supplement: CB-006-D5CB00060B-s001 [file CB-006-D5CB00060B-s001.pdf]

Supplementary Information (SI) for RSC Chemical Biology.
This journal is © The Royal Society of Chemistry 2025

Supplementary information for

Mechanistic Insights into the ATP-Mediated and Species-Dependent Inhibition of TrpRS by Chuangxinmycin

Authors: Yichen Ren[1], Sili Wang[1], Wen Liu[1*], Jing Wang[1,2*], Pengfei Fang[1,2*]

Affiliations:

[1]State Key Laboratory of Chemical Biology, Shanghai Institute of Organic Chemistry, University of Chinese Academy of Sciences, Chinese Academy of Sciences, 345 Lingling Road, Shanghai 200032, China

[2]School of Chemistry and Materials Science, Hangzhou Institute for Advanced Study, University of Chinese Academy of Sciences, 1 Sub-lane Xiangshan, Hangzhou 310024, China.

[*]To whom correspondences should be addressed. Email: wliu@sioc.ac.cn (W.L.), jwang@sioc.ac.cn (J.W.), or fangpengfei@sioc.ac.cn (P.F.).

This file contains:

Table S1–7

Figure S1–13

Table S1 Data collection and structure refinement statistics of *Ec*TrpRS/CXM/TrpAMP complex and *Sa*TrpRS/CXM complex.

**Dataset**	*Ec*TrpRS/CXM/TrpAMP	*Sa*TrpRS/CXM
**PDB code**	9KXR	9KWH
**Data collection**		
Space group	$P\ 1\ 2_1\ 1$	$C\ 1\ 2\ 1$
Cell dimensions		
a, b, c (Å)	62.11, 91.12, 75.08	184.60, 66.52, 99.50
α, β, γ (°)	90.00, 109.42, 90.00	90.00, 101.65, 90.00
Resolution (Å)	36.00~2.24(2.30~2.24)	30.23~2.38(2.44~2.38)
R_{merge}	0.145(0.813)	0.114(1.081)
Mean I/σ(I)	5.5(1.8)	10.8(1.7)
$CC_{1/2}$	0.982(0.901)	0.998(0.802)
Completeness (%)	100.0(100.0)	99.6(99.9)
Redundancy	6.3(5.2)	6.6(6.3)
**Refinement**		
Resolution (Å)	35.44~2.24	30.23~2.38
Unique reflections	37901	47385
R_{work} / R_{free} (%)	20.46/21.83	19.53/20.28
No. of atoms		
protein	5162	7513
ligand	53	48
solvent	224	130
B-factors		
protein	42.843	59.850
ligand	41.646	62.805
solvent	44.937	58.011
R. m. s. d.		
bond length (Å)	0.009	0.008
bond angle (°)	1.024	1.067
Rama. plot		
most favored (%)	96.68	95.98
additional allowed (%)	3.32	4.02

*Values in parentheses are for the highest-resolution shell.

Table S2 Data collection and structure refinement statistics of apo-*Sa*TrpRS and *Sa*TrpRS/Trp complex.

**Dataset**	apo-*Sa*TrpRS	*Sa*TrpRS/Trp
**PDB code**	9KVC	9KW0
**Data collection**		
Space group	*C* 1 2 1	*C* 1 2 1
Cell dimensions		
a, b, c (Å)	186.25, 69.71, 98.44	186.68, 66.93, 98.60
α, β, γ (°)	90.00, 101.68, 90.00	90.00, 101.72, 90.00
Resolution (Å)	91.20-3.04(3.20-3.04)	62.85-2.32(2.45-2.32)
R_{merge}	0.158(0.629)	0.116(0.662)
Mean I/σ(I)	9.3(2.6)	10.3(2.4)
$CC_{1/2}$	0.995(0.880)	0.997(0.924)
Completeness (%)	98.8(98.3)	99.1(99.5)
Redundancy	5.7(5.3)	6.5(6.8)
**Refinement**		
Resolution (Å)	65.12-3.04	62.85-2.32
Unique reflections	23678	51173
R_{work} / R_{free} (%)	20.93/22.97	19.81/21.58
No. of atoms		
protein	7504	7492
ligand	0	45
solvent	21	226
B-factors		
protein	60.598	48.731
ligand	0	54.139
solvent	60.740	48.014
R. m. s. d.		
bond length (Å)	0.010	0.008
bond angle (°)	1.253	1.016
Rama. plot		
most favored (%)	94.17	96.30
additional allowed (%)	5.72	3.70

*Values in parentheses are for the highest-resolution shell.

Table S3 Data collection and structure refinement statistics of two forms of *Ec*TrpRS/CXM/ATP complex structure.

**Dataset**	symmetric *Ec*TrpRS/CXM/ATP	asymmetric *Ec*TrpRS/CXM/ATP
**PDB code**	9M1F	9M1G
**Data collection**		
Space group	$P\ 1\ 2_1\ 1$	$P\ 1\ 2_1\ 1$
Cell dimensions		
a, *b*, *c* (Å)	63.42, 95.29, 64.66	62.01, 94.61, 67.34
α, β, γ (°)	90.00, 109.34, 90.00	90.00, 111.46, 90.00
Resolution (Å)	31.76-1.92(1.97-1.92)	26.12-2.15(2.21-2.15)
R_{merge}	0.111(0.534)	0.102(0.767)
Mean I/σ(I)	7.3(2.4)	10.9(1.9)
$CC_{1/2}$	0.989(0.785)	0.997(0.690)
Completeness (%)	99.2(91.5)	99.7(99.9)
Redundancy	5.7(3.4)	6.4(5.3)
**Refinement**		
Resolution (Å)	30.45-1.92	24.09-2.15
Unique reflections	54925	39235
R_{work} / R_{free} (%)	18.16/20.90	18.02/18.98
No. of atoms		
protein	5164	5128
ligand	96	96
solvent	564	343
B-factors		
protein	20.431	36.114
ligand	11.697	44.569
solvent	26.993	42.106
R. m. s. d.		
bond length (Å)	0.008	0.009
bond angle (°)	1.015	0.999
Rama. plot		
most favored (%)	97.72	97.57
additional allowed (%)	2.13	2.43

*Values in parentheses are for the highest-resolution shell.

Table S4 Data collection and structure refinement statistics of *Ec*TrpRS complexes with dCXM and mCXM.

**Dataset**	*Ec*TrpRS/dCXM/TrpAMP	*Ec*TrpRS/mCXM/TrpAMP
**PDB code**	9KY3	9KY9
**Data collection**		
Space group	$P\ 1\ 2_1\ 1$	$P\ 1\ 2_1\ 1$
Cell dimensions		
a, *b*, *c* (Å)	61.55, 90.43, 74.64	61.76, 90.85, 74.75
α, *β*, *γ* (°)	90.00, 109.26, 90.00	90.00, 109.47, 90.00
Resolution (Å)	58.11-2.25 (2.32-2.25)	70.48-2.19 (2.25-2.19)
R_{merge}	0.057(0.304)	0.126(0.489)
Mean I/σ(I)	20.1(5.0)	8.0(3.2)
$CC_{1/2}$	0.999(0.968)	0.926(0.748)
Completeness (%)	99.6(99.0)	100.0(99.8)
Redundancy	6.5(6.4)	6.1(4.7)
**Refinement**		
Resolution (Å)	58.11-2.25	70.48-2.19
Unique reflections	36687	39660
R_{work} / R_{free} (%)	18.69/20.43	20.81/23.69
No. of atoms		
protein	5176	5110
ligand	52	54
solvent	351	246
B-factors		
protein	37.859	39.721
ligand	31.770	35.155
solvent	41.798	41.108
R. m. s. d.		
bond length (Å)	0.009	0.008
bond angle (°)	1.029	0.994
Rama. plot		
most favored (%)	96.53	97.09
additional allowed (%)	3.47	2.91

*Values in parentheses are for the highest-resolution shell.

Table S5 Data collection and structure refinement statistics of *Sa*TrpRS complexes with dCXM and mCXM.

**Dataset**	*Sa*TrpRS/dCXM	*Sa*TrpRS/mCXM
**PDB code**	9KWW	9KXB
**Data collection**		
Space group	*C* 1 2 1	*C* 1 2 1
Cell dimensions		
a, b, c (Å)	183.94, 66.22, 99.31	184.95, 66.75, 100.17
α, β, γ (°)	90.00, 101.53, 90.00	90.00, 101.73, 90.00
Resolution (Å)	73.89-2.12 (2.18-2.12)	98.08-2.21 (2.27-2.21)
R_{merge}	0.093(0.766)	0.121(0.810)
Mean I/σ(I)	9.1(1.6)	6.5(1.6)
$CC_{1/2}$	0.994(0.598)	0.991(0.685)
Completeness (%)	99.7(99.2)	100.0(100.0)
Redundancy	6.1(4.9)	6.2(5.1)
**Refinement**		
Resolution (Å)	73.89-2.12	60.66-2.21
Unique reflections	66185	60034
R_{work} / R_{free} (%)	20.11/24.73	21.16/22.01
No. of atoms		
protein	7534	7517
ligand	45	51
solvent	435	282
B-factors		
protein	44.037	48.985
ligand	45.081	44.753
solvent	44.637	49.112
R. m. s. d.		
bond length (Å)	0.007	0.008
bond angle (°)	0.910	1.040
Rama. plot		
most favored (%)	96.21	95.46
additional allowed (%)	3.79	4.54

*Values in parentheses are for the highest-resolution shell.

Table S6 Sequence of primers used.

Primers		
**Name**	**Use**	**Sequence (5' to 3')**
EcWRS-1F	amplification of *Ec*TrpRS gene	ATGACTAAGCCCATCGTTTTTAGTGGCGCACAG
EcWRS-1Fa	amplification of *Ec*TrpRS gene for cloning	AGAAGGAGATATACCATGACTAAGCCCATCGTTTTTAGTGGCGC
EcWRS-1B	amplification of *Ec*TrpRS gene	CGATTGGTTTTGTGGCGAAGCCGCACCACCACCACCACCACTGAGATC
EcWRS-2F	amplification of pET-28a(+) vector for cloning	CGATTGGTTTTGTGGCGAAGCCGCACCACCACCACCACCACTGAGATC
EcWRS-2B	amplification of pET-28a(+) vector for cloning	GGCTTAGTCATGGTATATCTCCTTCTTAAAGTTAAACAAAATTATTTCTAGAG

Table S7 Open reading frame (ORF) sequences of genes.

Genes	
**Name**	**ORF Sequence (5’ to 3’)**
*Ec*TrpRS-6×His	ATGACTAAGCCCATCGTTTTTAGTGGCGCACAGCCCTCAGGTGAAT<br>TGACCATTGGTAACTACATGGGTGCGCTGCGTCAGTGGGTAAACAT<br>GCAGGATGACTACCATTGCATTTACTGTATCGTTGACCAACACGCG<br>ATCACCGTGCGCCAGGATGCACAGAAGCTGCGTAAAGCGACGCTG<br>GATACGCTGGCCTTGTATCTGGCTTGTGGTATCGATCCTGAGAAAA<br>GCACCATTTTTGTTCAGTCCCACGTACCAGAACATGCGCAGTTAGG<br>CTGGGCACTGAACTGCTATACCTACTTCGGCGAACTGAGCCGCATG<br>ACCCAGTTTAAAGATAAATCTGCGCGTTATGCCGAGAACATCAACG<br>CTGGTCTGTTTGACTATCCGGTGCTGATGGCTGCGGACATCCTGCTG<br>TATCAAACTAATCTGGTACCGGTGGGTGAAGACCAGAAACAGCAC<br>CTGGAACTGAGTCGCGATATCGCCCAGCGTTTCAACGCGCTGTATG<br>GCGAGATCTTTAAGGTGCCGGAGCCGTTTATTCCGAAATCTGGCGC<br>GCGCGTAATGTCGCTGCTGGAGCCGACCAAGAAGATGTCCAAGTCT<br>GACGATAACCGCAATAACGTTATCGGCCTGCTGGAAGATCCGAAAT<br>CGGTAGTGAAGAAAATCAAACGCGCGGTCACTGACTCCGACGAGC<br>CGCCGGTAGTTCGCTACGATGTGCAGAACAAAGCGGGCGTTTCCAA<br>CCTGCTGGATATCCTTTCTGCGGTAACGGGCCAGAGCATCCCGGAA<br>CTGGAAAAACAGTTCGAAGGCAAGATGTATGGTCATCTGAAAGGT<br>GAAGTGGCTGATGCCGTTTCCGGTATGCTGACTGAATTGCAGGAAC<br>GCTATCACCGTTTCCGCAACGATGAAGCCTTCCTGCAACAAGTGAT<br>GAAAGATGGCGCGGAAAAAGCCAGCGCGCACGCTTCCCGTACGCT<br>AAAAGCGGTATACGAAGCGATTGGTTTTGTGGCGAAGCCGCACCA<br>CCACCACCACCACTGA
*Sa*TrpRS-6×His	ATGGAAACTCTGTTCTCCGGTATCCAGCCGTCCGGTATTCCGACCA<br>TCGGTAACTACATCGGTGCGCTGAAACAGTTCGTGGACGTACAGAA<br>CGACTACGACTGCTACTTCTGCATCGTTGATCAGCACGCTATCACT<br>ATGCCACAAGATCGTCTGAAACTGCGTAAACAGACTCGTCAGCTGG<br>CAGCGATCTACCTGGCATCTGGTATCGATCCGGACAAAGCTACTCT<br>GTTCATCCAGTCTGAAGTTCCGGCTCACGTGCAGGCAGGCTGGATG<br>CTGACCACCATCGCGTCTGTTGGCGAACTGGAACGTATGACTCAGT<br>ACAAAGACAAAGCACAGAAAGCGGTTGAAGGTATCCCGGCAGGCT<br>TGCTGACCTACCCGCCATTGATGGCGGCTGATATCGTGCTGTACAA<br>CACCAACATCGTTCCAGTTGGTGACGACCAGAAACAGCATATCGAA<br>CTGACTCGTAACCTGGTTGACCGTTTCAACTCTCGTTACAACGACGT<br>GCTGGTTAAACCAGAAATCCGTATGCCGAAAGTTGGTGGTCGTGTT<br>ATGTCTCTGCAAGATCCGACTCGTAAGATGTCCAAATCCGATGACA<br>ACGCGAAGAACTTCATCAGCCTGCTGGACGAACCGAACGTTGCAG<br>CGAAGAAGATCAAATCTGCTGTAACCGATTCTGACGGTATCATCAA<br>ATTCGATCGTGATAACAAACCGGGTATCACCAACCTGATCTCTATC<br>TACGCAGGTCTGACCGATATGCCGATCAAAGACATCGAAGCGAAA<br>TACGAAGGTGAAGGTTACGGCAAATTCAAAGGTGACCTGGCGGAA

	ATCGTTAAAGCATTCTTGGTGGAATTTCAAGAGAAATACGAATCTTTCTACAACTCCGATAAACTGGACGACATCTTGGATCAGGGTCGTGACAAAGCGCACAAAGTTAGCTTCAAGACCGTTAAGAAGATGGAGAAAGCGATGGGTCTGGGCCGTAAACGTCACCACCACCACCACCACTGA

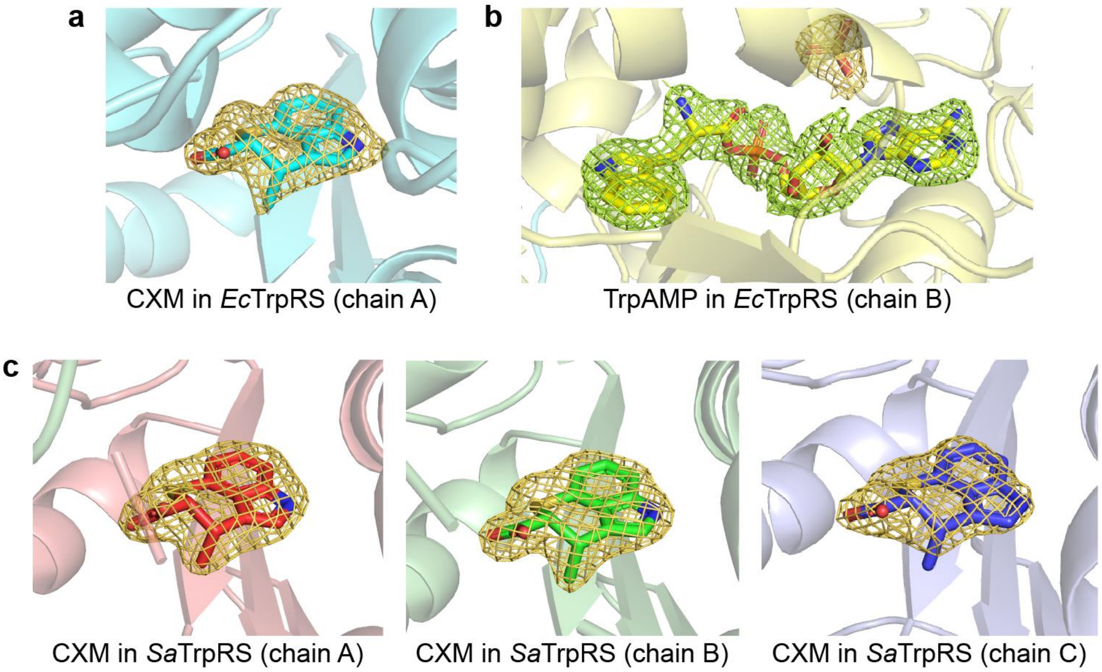

Fig. S1 Electron-density map ($2F_o - F_c$ map at 1.0 σ) of CXM and TrpAMP in *Ec*TrpRS and *Sa*TrpRS complexes. (a) CXM in *Ec*TrpRS/CXM complex (chain A). (b) TrpAMP and sulfate ion in *Ec*TrpRS/TrpAMP complex (chain B). (c) CXM in *Sa*TrpRS/CXM complex.

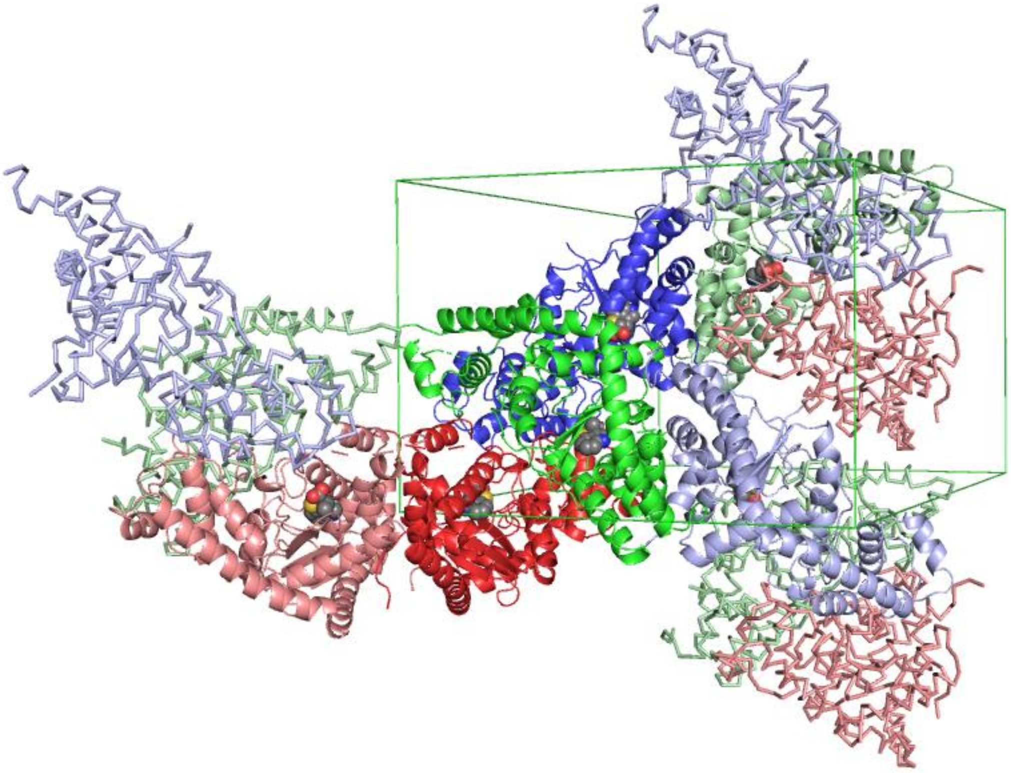

Fig. S2 Relationship between the asymmetric unit (chains A, B, and C shown in red, green, and blue, respectively) and adjacent asymmetric units (chains A, B, and C shown in salmon, pale green, and light blue, respectively) in the *Sa*TrpRS/CXM complex. The dimer formed by chains B and C exhibits non-crystallographic symmetry, while chain A forms a dimer with a crystallographically symmetric equivalent chain A. The unit cell of crystal shown as green lattice.

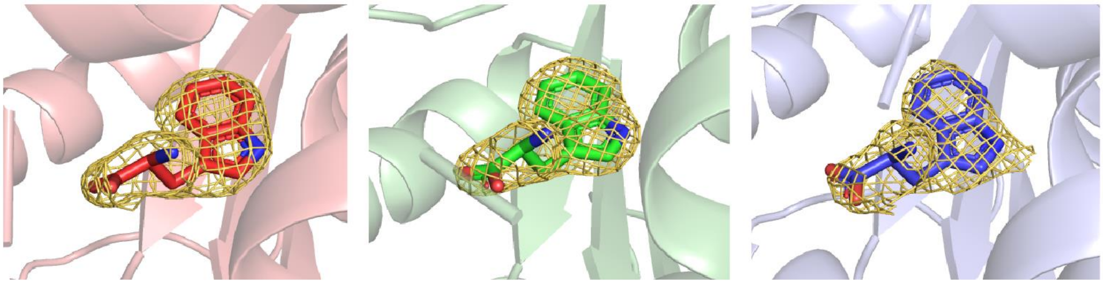

Fig. S3 Electron-density map ($2F_o - F_c$ map at 1.0 σ) of Trp in *Sa*TrpRS/Trp complex.

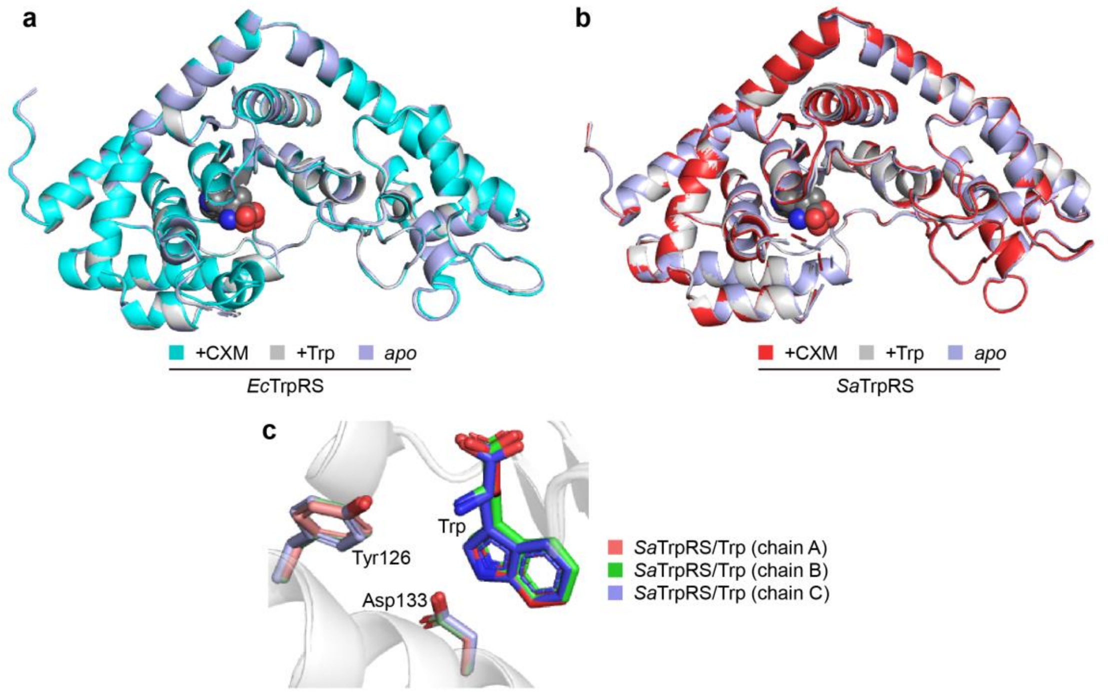

Fig. S4 (a) The *Ec*TrpRS/CXM (cyan), *Ec*TrpRS/Trp (gray), and apo-*Ec*TrpRS (pale blue) structures all adopt a similar open conformation. (b) The *Sa*TrpRS/CXM (red), *Sa*TrpRS/Trp (gray), and apo-*Sa*TrpRS (pale blue) structures also adopt a similar open conformation, with chain A displayed for each structure. (c) The three chains (A, B, and C) of the *Sa*TrpRS/Trp complex are nearly identical in the Trp-binding pocket. Key residues Y126 and D133, along with the bound Trp molecules, are displayed as sticks, while the main chains are represented as gray cartoons. Notably, Y126 adopts an "open-gate" conformation.

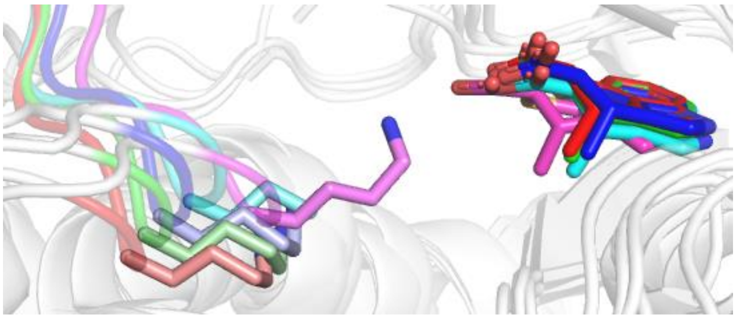

Fig. S5 The structures of *Ec*TrpRS/CXM, *Sa*TrpRS/CXM (chains A, B, and C), and the pre-transition state (PreTS) *Gs*TrpRS/CXM (PDB: 7CMS, chain B) were superimposed. The main chains of the KMSKS motif in these structures are colored cyan, red, green, blue, and magenta, respectively. The residue K195 in *Gs*TrpRS (and its equivalents K198 in *Ec*TrpRS and K196 in *Sa*TrpRS), along with the bound CXM molecules, are displayed as sticks.

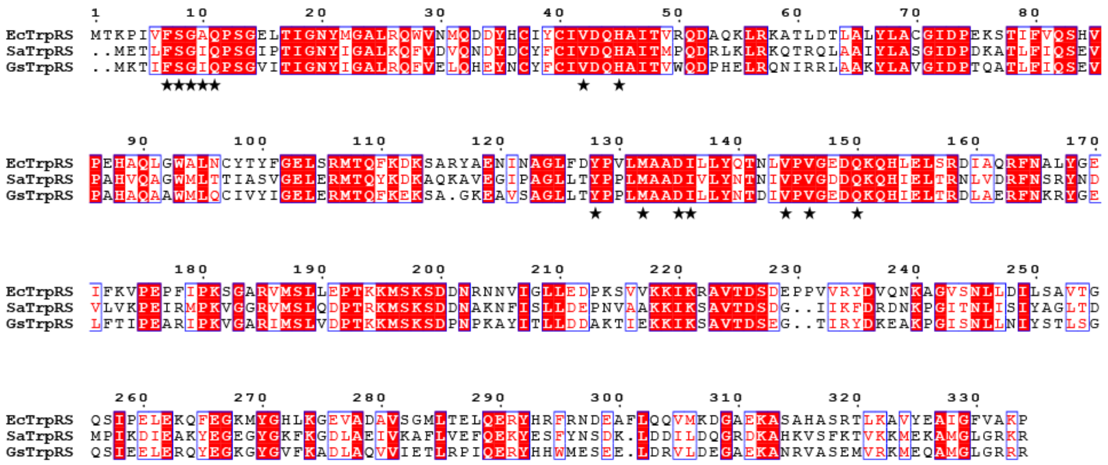

Fig. S6 Sequence alignment of *Ec*TrpRS (UniProt: P00954), *Sa*TrpRS (UniProt: P67594) and *Gs*TrpRS (UniProt: P00953). The residues located within 4 Å of the ligand skeleton in CXM complexes are marked with asterisks. Residues highlighted in red shading are identical.

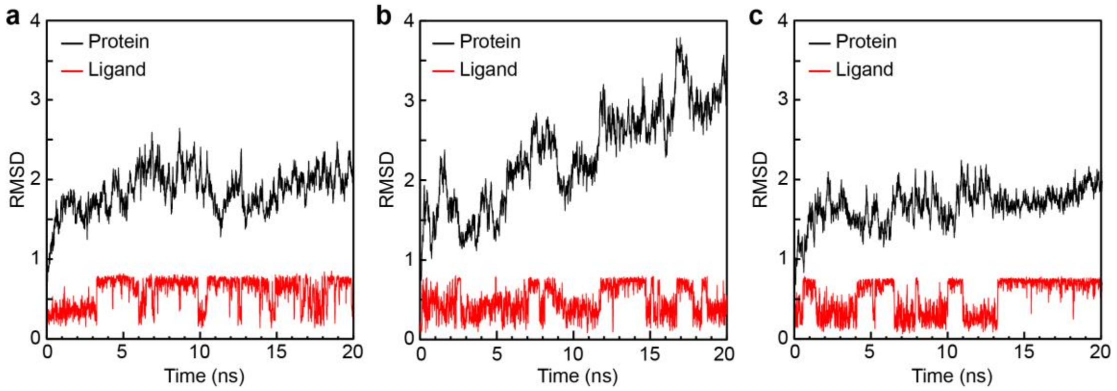

Fig. S7 Time evolution of RMSD of protein main chain atoms and CXM heavy atoms during MD simulation (10 ps/frame, reference: the last frame of previous phase). (a) *Ec*TrpRS/CXM system. (b) The first 20 ns phase of *Sa*TrpRS/CXM system. (c) The continued 20 ns phase of *Sa*TrpRS/CXM system.

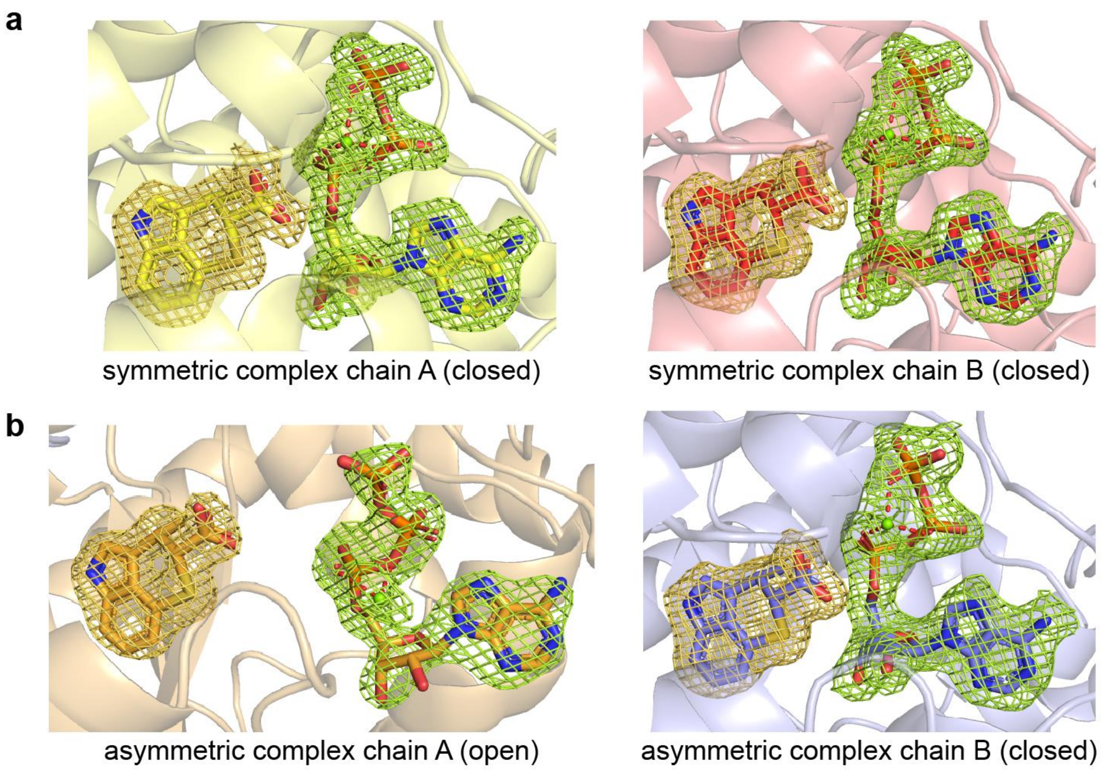

Fig. S8 Electron-density map ($2F_o - F_c$ map at 1.0 σ) of CXM and ATP in *Ec*TrpRS/CXM/ATP complexes. (a) Symmetric *Ec*TrpRS/CXM/ATP complex. (b) Asymmetric *Ec*TrpRS/CXM/ATP complex.

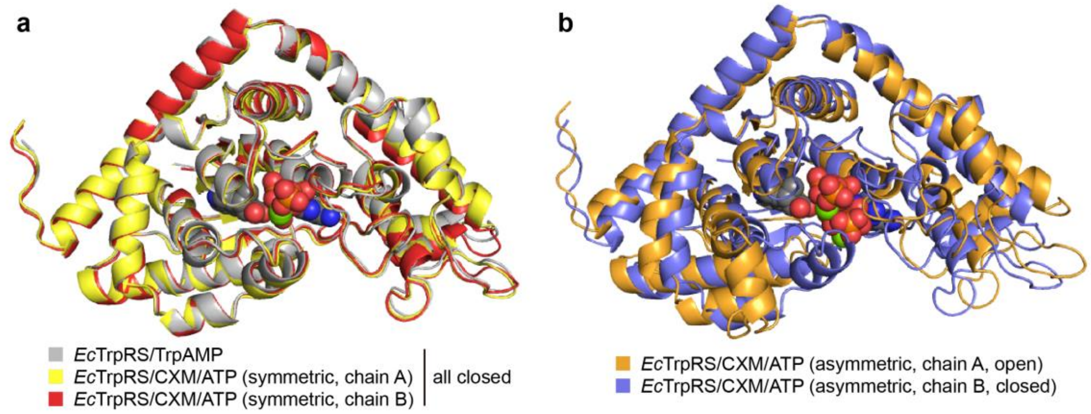

Fig. S9 Conformational comparison of the two chains in *Ec*TrpRS/CXM/ATP complexes. (a) Symmetric structure (chain A in yellow, chain B in red) superimposed with the closed-state *Ec*TrpRS/TrpAMP complex (gray, PDB: 8I1W chain B). (b) Superposition of chain A (orange) and chain B (slate) from the asymmetric structure.

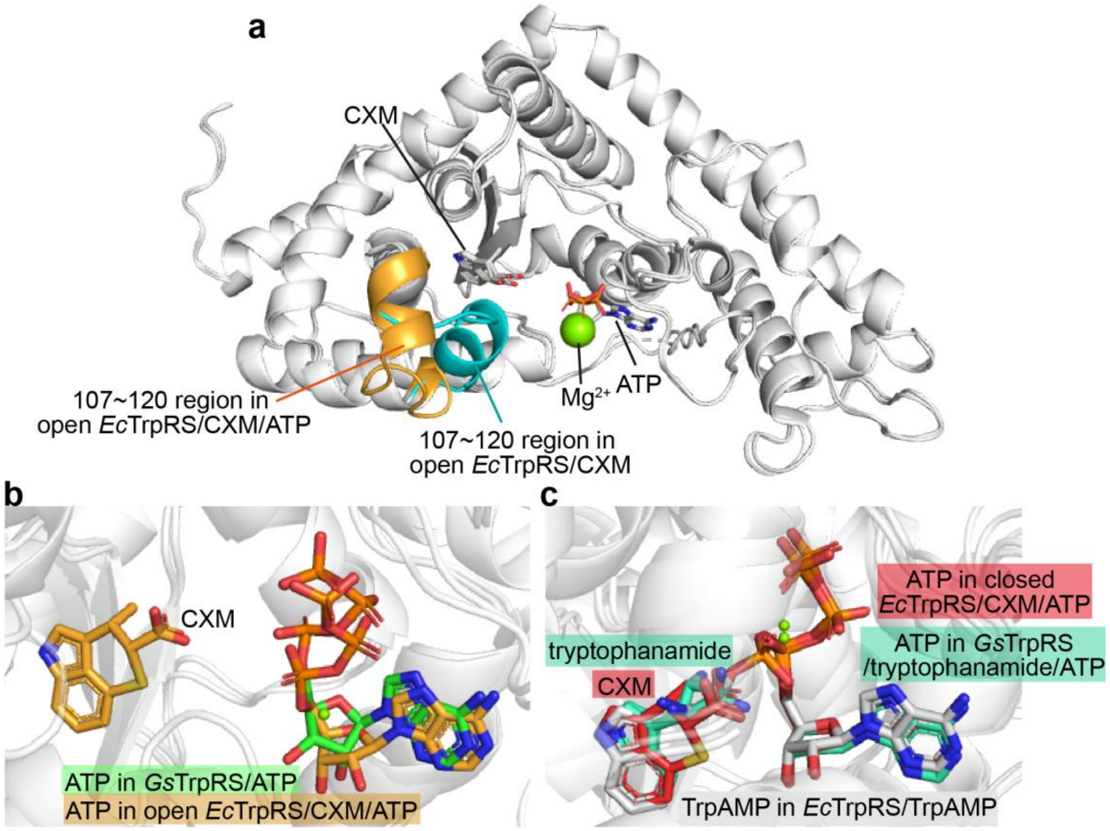

Fig. S10 Structural comparison of *Ec*TrpRS/CXM/ATP complex with other TrpRS complexes. (a) Superimposition of the open-state *Ec*TrpRS/CXM/ATP and *Ec*TrpRS/CXM structures. Well-aligned regions are shown as gray cartoons, while the conformationally divergent loop 107–120 is highlighted in orange (*Ec*TrpRS/CXM/ATP) and cyan (*Ec*TrpRS/CXM). (b) Comparison of ATP binding between the open-state *Ec*TrpRS/CXM/ATP structure and the open-state *Gs*TrpRS/ATP structure (PDB: 1MAW, chain A). Superimposed proteins are displayed as gray cartoons. *Ec*TrpRS-bound ligands are shown as orange sticks, and *Gs*TrpRS-bound ligands as green sticks. (c) Superimposition of the closed-state *Ec*TrpRS/CXM/ATP structure, *Gs*TrpRS/tryptophanamide/ATP structure (PDB: 1MAU), and *Ec*TrpRS/TrpAMP structure (PDB: 8I1W, chain B). *Ec*TrpRS-bound CXM and ATP are shown as red sticks, *Gs*TrpRS-bound ligands as cyan sticks, and *Ec*TrpRS-bound TrpAMP as gray sticks.

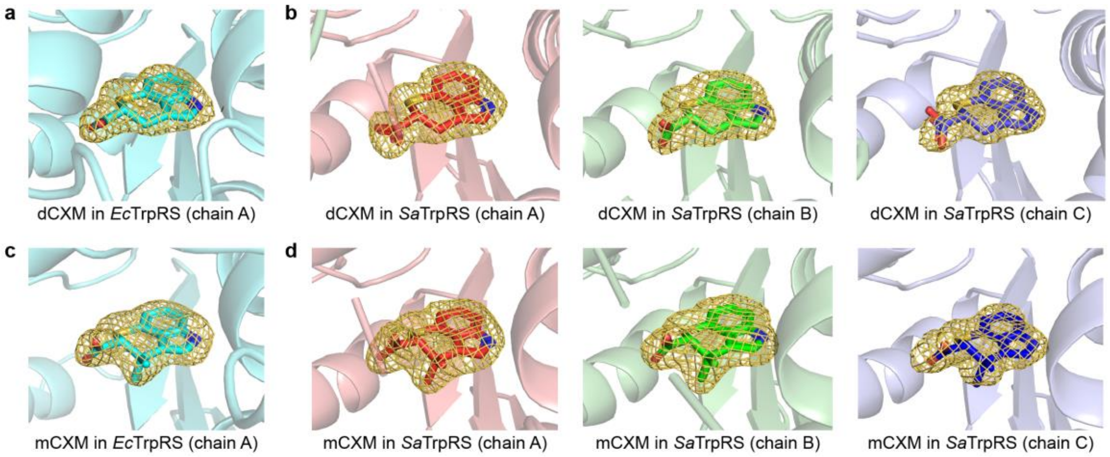

Fig. S11 Electron-density map ($2F_o - F_c$ map at 1.0 σ) of dCXM and mCXM in *Ec*TrpRS and *Sa*TrpRS complexes. (a) dCXM in *Ec*TrpRS/dCXM complex (chain A). (b) dCXM in *Sa*TrpRS/dCXM complex. (c) mCXM in *Ec*TrpRS/mCXM complex (chain A). (d) mCXM in *Sa*TrpRS/mCXM complex.

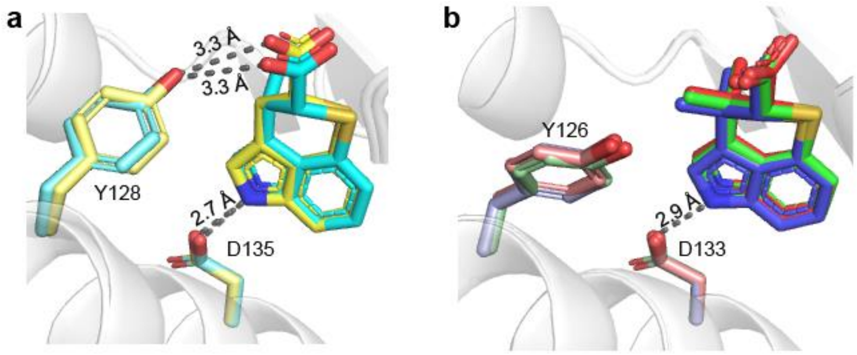

Fig. S12 Binding of dCXM and mCXM. (a) Comparison of *Ec*TrpRS complexed with dCXM (yellow) and CXM (cyan). (b) mCXM binding in three chains (red, green and blue respectively) of *Sa*TrpRS/mCXM complex.

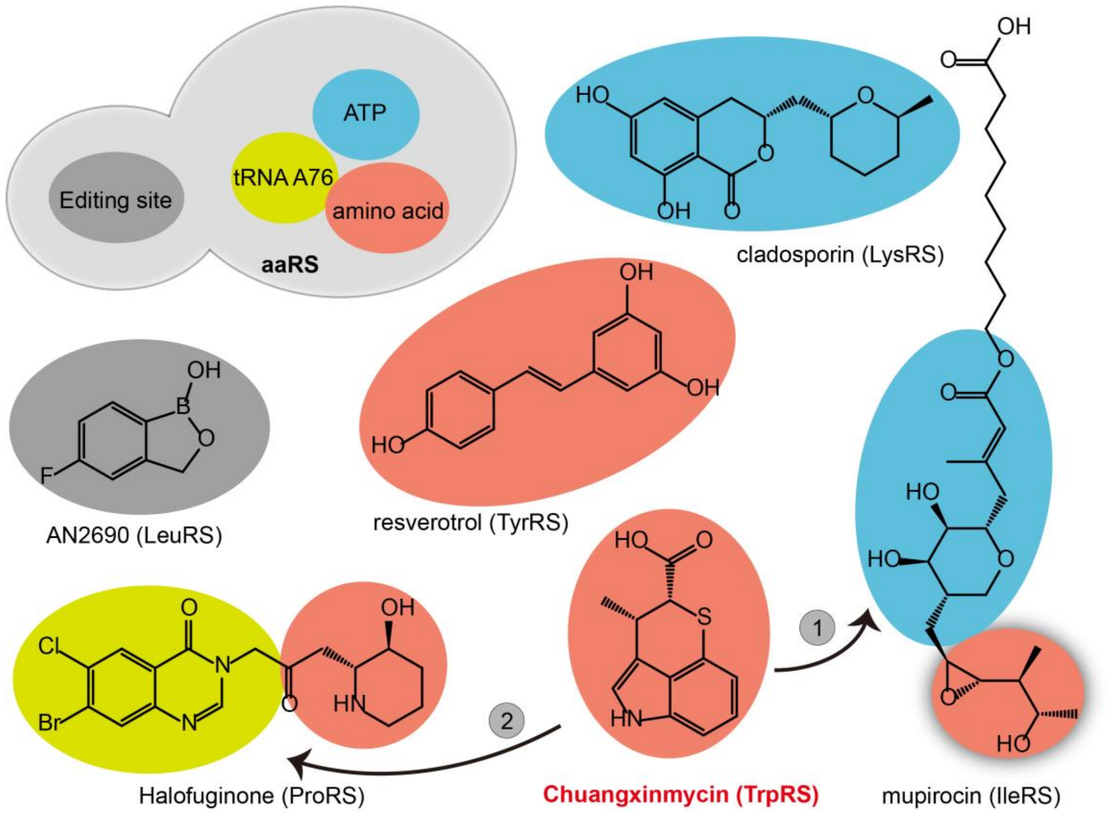

Fig. S13 Chuangxinmycin (CXM) is a unique single-site inhibitor targeting aminoacyl-tRNA synthetase (aaRS). The active center of aaRS contains several key binding sites: the amino acid binding site (red oval), the ATP binding site (blue oval), the tRNA A76 binding site (yellow oval), and in some aaRSs, an editing site (grey oval). These sites are primary targets for aaRS inhibitors. CXM and other typical aaRS inhibitors are highlighted in corresponding colors to indicate their binding locations. Enhancing inhibitor potency could be achieved through strategic modifications of CXM derivatives to access either the ATP-binding pocket or tRNA A76-binding site. The targeted aaRSs are indicated in parentheses following each compound.
